# Advancing therapeutic strategies for graft-versus-host disease by targeting gut microbiome dynamics in allogeneic hematopoietic stem cell transplantation: current evidence and future directions

**DOI:** 10.1186/s10020-024-01060-x

**Published:** 2025-01-03

**Authors:** Muhammad Azhar Ud Din, Yan Lin, Changkun Lyu, Chengxue Yi, Anning Fang, Fei Mao

**Affiliations:** 1 Department of Laboratory Medicine, The Affiliated People’s Hospital, Jiangsu University, No. 8 Dianli Road, Zhenjiang, 212002 Jiangsu People’s Republic of China; 2 Institute of Hematology, Jiangsu University, Zhenjiang, 212013 Jiangsu People’s Republic of China; 3 The People’s Hospital of Danyang, Affiliated Danyang Hospital of Nantong University, Zhenjiang, 212399 Jiangsu People’s Republic of China; 4 School of Medical Technology, Shangqiu Medical College Shangqiu, Shangqiu, 476100 Henan People’s Republic of China; 5 School of Medical Technology, Zhenjiang College, Zhenjiang, 212028 Jiangsu People’s Republic of China; 6 Basic Medical School, Anhui Medical College, 632 Furong Road, Economic and Technological Development Zone, Hefei, 230061 Anhui People’s Republic of China

**Keywords:** Intestinal bacteria, Allogeneic hematopoietic stem cell transplantation, Graft-versus-host disease

## Abstract

Hematopoietic stem cell transplantation (HSCT) is a highly effective therapy for malignant blood illnesses that pose a high risk, as well as diseases that are at risk due to other variables, such as genetics. However, the prevalence of graft-versus-host disease (GVHD) has impeded its widespread use. Ensuring the stability of microbial varieties and associated metabolites is crucial for supporting metabolic processes, preventing pathogen intrusion, and modulating the immune system. Consequently, it significantly affects the overall well-being and susceptibility of the host to disease. Patients undergoing allogeneic hematopoietic stem cell transplantation (allo-HSCT) may experience a disruption in the balance between the immune system and gut bacteria when treated with medicines and foreign cells. This can lead to secondary intestinal inflammation and GVHD. Thus, GM is both a reliable indicator of post-transplant mortality and a means of enhancing GVHD prevention and treatment after allo-HSCT. This can be achieved through various strategies, including nutritional support, probiotics, selective use of antibiotics, and fecal microbiota transplantation (FMT) to target gut microbes. This review examines research advancements and the practical use of intestinal bacteria in GVHD following allo-HSCT. These findings may offer novel insights into the prevention and treatment of GVHD after allo-HSCT.

## Highlights

1. Hematopoietic stem cell transplantation (HSCT) is a powerful treatment for high-risk blood diseases and genetic disorders.
2. Graft-versus-host disease (GVHD) remains a major obstacle to the broader application of HSCT.
3. Maintaining a stable gut microbiome is crucial for supporting metabolism, preventing infections, and regulating the immune system.
4. Allogeneic HSCT can disrupt the gut-immune balance, causing inflammation and triggering GVHD.
5. Innovative strategies like nutritional support, probiotics, selective antibiotics, and fecal microbiota transplantation (FMT) can prevent and treat GVHD post-allo-HSCT.

## Introduction

Allogeneic hematopoietic stem cell transplantation (allo-HSCT) is widely recognized as a potentially curative treatment for both malignant and non-malignant hematologic disorders (Loke et al. 2020). However, there is often a high rate of morbidity and mortality following this treatment, primarily due to complications such as infections and GVH (Michonneau et al. 2024; Sahin et al. 2016). In patients with GVHD, immune-competent T cells from the transplant attack the recipient's cells as they proliferate in the immunocompromised host, severely impairing organ function (Ghimire et al. 2017). Recent studies indicate that the microbiome plays a crucial role in the development of GVHD after allogeneic hematopoietic stem cell transplantation and influences treatment outcomest (Hong et al. 2021). The conditioning regimen for allo-HSCT can damage mucosal epithelial surfaces, leading to gut bacterial translocation and disrupting microbial balance (Chang et al. 2021). Additionally, the routine use of antibiotics for gut decontamination further alters the intestinal flora, impacting both aerobic and anaerobic bacteria that are involved in the pathophysiology of GVH (Murphy and Nguyen 2011; Staffas et al. 2017). Evidence suggests that the type of antibiotics used and the timing of their administration before to, during, or following transplantation may have an impact on microbiome diversity and, consequently, long-term clinical outcomes (Shono and van den Brink 2017). High-capacity sequencing studies have provided detailed insights into the composition and diversity of microorganisms present in the gut (Gao et al. 2021; Taur et al. 2014). High-capacity sequencing studies have provided detailed insights into the composition and diversity of microorganisms present in the gut (Henig et al. 2021; Peled et al. 2020).

The mammalian gut microbiome is composed of a complex and dynamic array of bacterial colonies, fungi, archaea, viruses, and other microorganisms. It is estimated that the density of these microorganisms in the gut ranges from 1 × 10^13^ to 1 × 10^14^ per gram of feces (Chen et al. 2021). Among these, anaerobic bacteria comprise approximately 51% of the total, while bacterioid bacteria comprise approximately 48%. The remaining 1% included Proteus, Microcystins, Clostridium, Cyanobacteria, Actinomyces, Spirulina, numerous fungi, protozoa, viruses, and other microorganisms (Pennycook and Scanlan 2021). Symbiotic microorganisms influence the overall well-being and susceptibility of the body to diseases by enhancing metabolic processes, defending against harmful pathogens, and maintaining the immune system's balance. Symbiotic and pathogenic bacteria collaborate to sustain the dynamic equilibrium of intestinal microbes under symbiotic conditions. When patients undergoing allo-HSCT receive chemotherapy, radiation, antibiotics, or other treatments, either separately or in combination, the delicate equilibrium between the microbiota and host immune system is significantly disrupted (Khuat et al. 2020). The gut microbiota plays a crucial and intricate role in developing GVHD. This review specifically examines the impact of the intestinal microbiome on the development of GVHD. It discusses potential strategies before and after transplantation to maintain a healthy microbiome and use gut flora-targeted therapies to prevent or treat GVHD. The aim is to provide comprehensive insights for future clinical research and application.

### Digestive tract manifestations of GVHD after hematopoietic stem cell transplantation

GVHD following allogeneic allo-HSCT is a significant cause of morbidity and non-relapse mortality (NRM). Gastrointestinal (GI) complications are common and can be severe manifestations of GVHD. This condition can disrupt various parts of the gastrointestinal tract, with the cecum, ileum, and ascending colon being the most frequently affected areas. Additionally, GVHD can also impact the stomach, duodenum, and rectum (Scott et al. 2018). From a clinical standpoint, gastrointestinal graft-versus-host disease (GI-GVHD) typically presents with symptoms such as nausea, vomiting, loss of appetite, abdominal cramping, and weight loss. Severe involvement of the lower gastrointestinal tract may manifest as stomach cramps and diarrhea, which can be either watery or bloody (Wang et al. 2022). Diagnostic methods, including gastrointestinal endoscopy and histological analysis, are essential for confirming GI-GVHD based on clinical symptoms. Key endoscopic findings include the presence of mucosal crypts, glandular hyperplasia, crypt abscesses, and obstructions, which serve as important indicators of GI-GVHD. Pathological features that support the diagnosis include ulceration, shedding of the epithelium, gland apoptosis, and apoptosis of epithelial cells (Naymagon et al. 2017). Additionally, imaging techniques such as ultrasound, CT scans, MRI, and PET-CT can enhance diagnostic accuracy (Derlin et al. 2015; Spadea et al. 2022). However, there are currently no standardized criteria for diagnosing GI-GVHD.

### Effects of GVHD on the upper gastrointestinal tract

Symptoms of upper GI-GVHD primarily affect the stomach and esophagus. Esophageal GVHD often presents as difficulty swallowing (dysphagia) or chest discomfort (Trabulo et al. 2015). Endoscopic examinations typically reveal mucosal inflammation, atrophy of epithelial cells, redness, and erosion (Zhang et al. 2022). Diagnostic features include programmed cell death of epithelial cells and an increased number of lymphocytes within the epithelial layer (Länger et al. 2011). GI-GVHD may initially present with mild symptoms, and endoscopic findings can range from slight redness and swelling of the mucosa to severe erosion, ulceration, atrophy, and shedding of the mucosal layer (Ponec et al. 1999). When identified, mucosal exfoliation in the stomach has a 100% positive predictive value (PPV), although its specificity is only 18% due to its rarity. Gastric biopsies generally show apoptosis, gland destruction, limited inflammatory cell infiltration, and granular eosinophil debris. There is a strong correlation between histological findings and clinical grades of GVHD in the upper gastrointestinal tract, with clinical grading demonstrating high accuracy in predicting non-relapse mortality (NRM) (Sarraf et al. 2022).

### Effects of GVHD on the lower gastrointestinal tract

The lower gastrointestinal tract is particularly susceptible to GVHD, which can lead to serious complications such as protein-losing enteropathy, bloody diarrhea, hypoalbuminemia, and bowel obstruction (Robak et al. 2017). For lower GI-GVHD, endoscopic findings such as erosion, redness, edema, villous atrophy, and extensive ulcerative lesions are key diagnostic indicators. Although mucosal exfoliation is not commonly observed, its presence is a reliable sign of severe GVHD (Nomura et al. 2017). Biopsy results tend to be more specific than endoscopic findings and show a stronger correlation with histological grades and clinical severity (Yip et al. 2021). Histological analysis can identify GVHD lesions in mucosal areas that appear normal during endoscopy. Important histological features include clonal apoptosis and a reduction in lamina propria lymphocytes (Farooq et al. 2022). Other potential histological findings may include mucosal ulcers, atrophy, glandular dilation, localized fibrosis, and crypt abscesses. Therefore, histopathological analysis is crucial for the identification and classification of GVHD in various regions of the intestine.

## Intestinal epithelial cells’ protective effect against GI-GVHD

The intestinal epithelial tissue is composed of several functional cell types, including Paneth cells, intestinal stem cells, intestinal epithelial cells, and goblet cells (Gehart and Clevers 2019). Intestinal epithelial cells (IECs) play a vital role in nutrient absorption and form a physical barrier through tight junctions that separate the intestinal wall from the lumen. Secretory cells, such as Paneth and goblet cells, are essential for maintaining the balance of intestinal microbiota, ensuring homeostasis between the immune system and intestinal flora, and protecting the host from infections (Pelaseyed et al. 2014). ISCs, located near the base of the crypts, have the ability to differentiate into any cell type within the intestinal epithelium. Goblet cells contribute to the intestinal barrier by forming tight connections and secreting mucus, which protects IEC (Levine et al. 2013; Petersson et al. 2011) The glycoprotein MUC2 is the primary component of intestinal mucus, creating a thick barrier that is largely impermeable to most intestinal bacteria (Birchenough et al. 2015). However, certain intestinal bacteria can metabolize MUC2 glycans, potentially altering the bacterial composition and affecting the integrity of the mucus layer (Shono et al. 2016). Damage to goblet cells and changes in intestinal flora in patients with GVHD can disrupt the mucosal barrier, facilitating the translocation of pathogens and exacerbating both GVHD and infections.

**Fig. 1:**
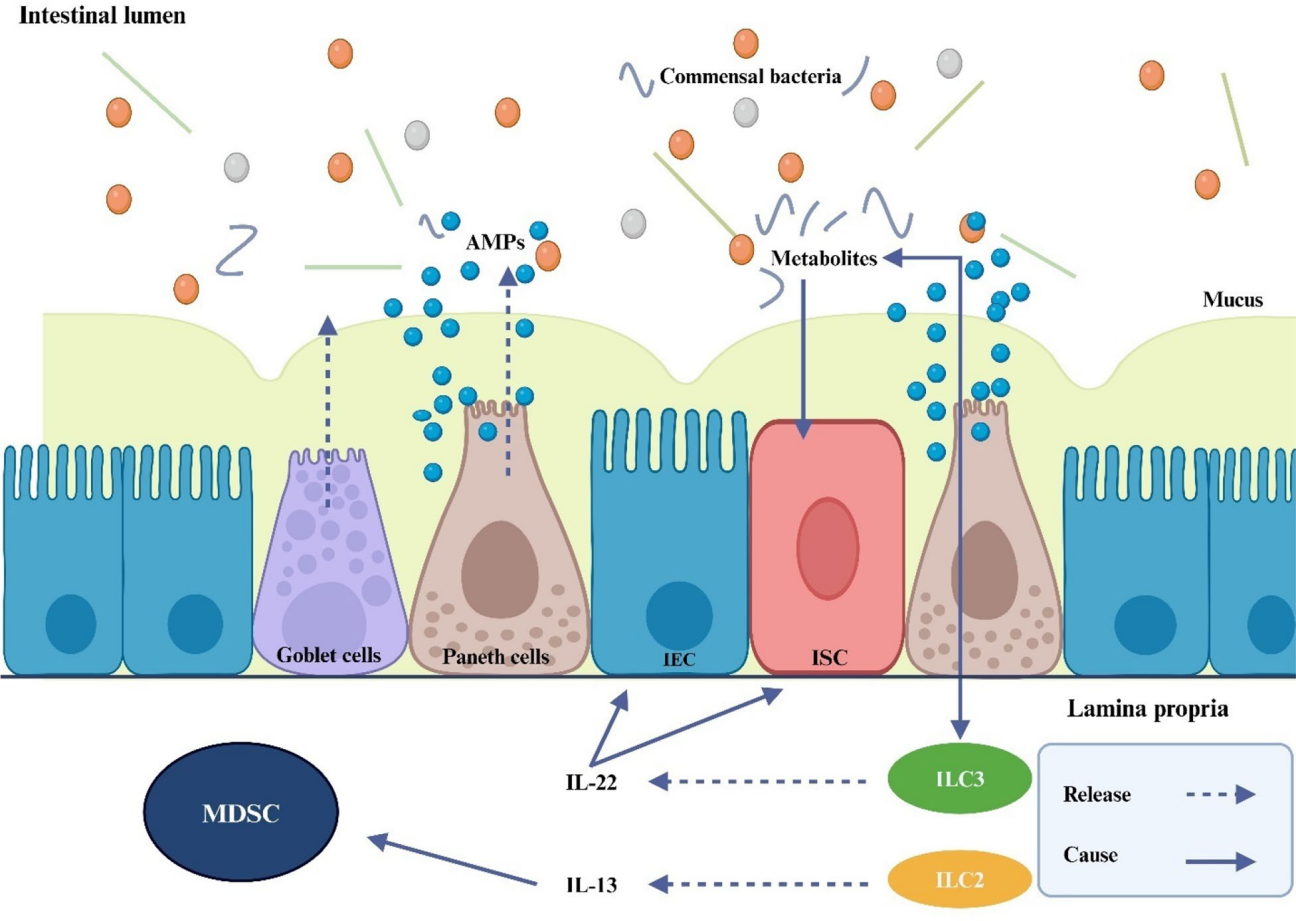
The host immune system inhiits the growth of infections and preserves the variety of the gut microbiota under physiological settings. IEC uphold a whole barrier on their surface to stop bacteria from migrating into the host tissues. Antimicrobial peptides (AMPs) are secreted into the intestinal lumen by Paneth cells in order to shield IECs from possible pathogens and intestinal microbiome bacteria. Moreover, AMPs supply ISC with nourishment signals and preserve the balance of microbes in the gut. By secreting mucin, goblet cells strengthen the barrier that divides microbiota from host tissue, further shielding the intestinal epithelium from lumen bacteria. Furthermore, symbiotic bacteria induce IL-22 production from innate lymphocytes (ILC) 3 in the lamina propria, which in turn induces Paneth cells to secrete AMP and sustain ISC. Because symbiotic bacteria's metabolites control intestinal inflammation and immunology, they can help lessen GI-GVHD and IEC damage. By generating IL-13, ILC2 encourages the growth of donor myeloid-derived suppressor cells (MDSC)

Paneth cells, located at the base of the small intestine's crypts, provide both chemical and physical barriers that prevent microorganisms from entering the crypt environment (Peterson and Artis 2014). These cells help maintain microbial diversity and homeostasis by secreting various antimicrobial peptides (AMPs), including lysozyme, intestinal α-defensins, secretory phospholipase A2 (PLA2), and regenerative islet-derived protein 3α (REG3α) (Ostaff et al. 2013). Paneth cells also support ISC activity by producing growth factors such as EGF, TGFα, Wnt3, and Notch ligands, acting as protectors of the intestinal crypts. The extent of Paneth cell loss in GVHD is directly correlated with the severity of the disease and the risk of non-relapse mortality. A reduction in Paneth cells leads to decreased secretion of AMPs, resulting in an imbalance of intestinal flora and the emergence of pathogenic bacteria. This disruption damages the protective barrier of the mucosal epithelium and activates antigen-presenting cells, further worsening GVHD (Eriguchi et al. 2012). The mechanism is illustrated in the Fig. 1.

**Fig. 2:**
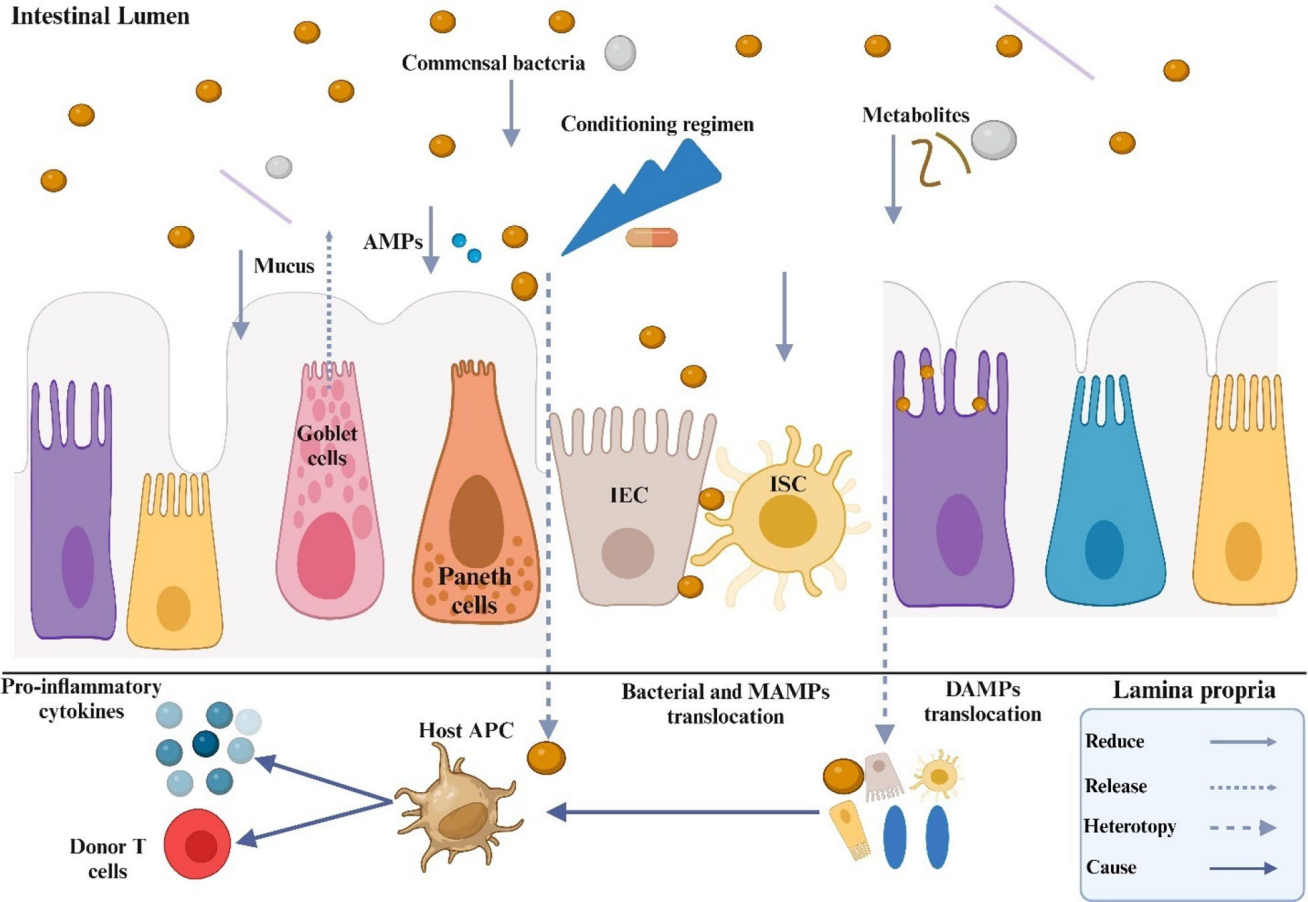
Cytotoxic preconditioning and GI-GVHD harm goblet cells, compromising the intestinal barrier and lowering mucosal barrier function. Paneth cell injury reduces AMP secretion and ISC niche function, while antibiotics and nutritional deficiency cause ecological imbalances and detrimental translocations that increase GVHD and pro-inflammatory responses

Intestinal homeostasis and regeneration following injury rely on intestinal stem cells (ISCs), which are located among Paneth cells at the base of the crypts. However, intestinal graft-versus-host disease (GVHD) specifically targets ISCs, making them a primary focus of damage and closely linked to the disease's pathogenesis. Implementing protective strategies for ISCs could provide significant benefits (Takashima et al. 2011). The impairment of Paneth and goblet cells exacerbates changes in the intestinal microbiota and GVHD, allowing pathogen-associated molecular patterns (PAMPs) to cross the intestinal barrier and activate the immune response. Monocytes recruit macrophages and neutrophils to inflamed areas, promoting the development of Th17 cells (Reinhardt et al. 2014), as shown in Fig. 2. Neutrophils generate reactive oxygen species, which can damage the intestinal barrier and surrounding tissues. They subsequently migrate to mesenteric lymph nodes to participate in allogeneic antigen presentation (Hülsdünker et al. 2018). Activated allogeneic donor T cells further contribute to the progression of GVHD.

The presence of innate lymphoid cells (ILCs) near the crypts is crucial for the proper functioning of the innate immune system. ILC2 and ILC3, located in the lamina propria of the small intestine and colon, do not have antigen-specific receptors but are important for maintaining immune balance (Björkström et al. 2013) ILC3 cells express major histocompatibility complex (MHC) class II, which helps to promote tolerance towards symbiotic bacteria and restricts excessive activation of T cells during infection. This ultimately leads to a reduction in intestinal immunity and inflammation (Hepworth et al. 2013; Qiu et al. 2013). ILC3 also secretes IL-22, which stimulates Paneth cells to produce REG3α and supports the regeneration of the intestinal epithelium through processes mediated by ISCs (Lindemans et al. 2015). Consequently, ILC3 plays a significant role in controlling inflammation during GVHD and helps preserve ISC function after injury (Aparicio-Domingo et al. 2015). ILC2 enhances survival rates in individuals who have undergone allo-HSCT, but its numbers decline in the intestinal lamina propria during GVHD (Yue et al. 2024). Infusing exogenous ILC2 can reduce the activation of pro-inflammatory Th1 and Th17 cells from the donor and increase the presence of myeloid-derived suppressor cells (MDSCs) in the gut by producing IL-13. ILCs are vital for protecting the gut against harmful microorganisms, promoting lymphoid tissue development, and supporting the body's anti-inflammatory and immune responses. As a result, they contribute to maintaining intestinal balance and stability while mitigating damage caused by GVHD to the gastrointestinal tract (Bruce et al. 2021).

## The role of gut microbiota in modulating MHC expression and GVHD

To enable T cells to identify and react to foreign antigens, MHC molecules are necessary for antigen presentation. MHC class II molecules on enterocytes have the ability to expose recipient T cells to donor-derived antigens, triggering an immunological response that results in GVHD. The gut microbiota has been found to affect the expression of MHC class II on enterocytes. The expression of these molecules, for example, might be enhanced or suppressed by particular microbial populations, which can alter the immune response (Koyama et al. 2023). According to one study, intestinal epithelial cells' expression of MHC class II can be increased by the presence of specific gut bacteria. Increased antigen presentation from this upregulation may make it more likely for donor T cells to identify recipient tissues as foreign, which could accelerate the development of aGVHD (Yeh et al. 2024). By lowering the quantity of APCs available, other microbial communities may, on the other hand, suppress MHC expression, which could have a protective impact against the onset of GVHD. The gut microbiome affects MHC expression through a variety of methods. One theory is that when dietary fibers are fermented, gut bacteria produce short-chain fatty acids (SCFAs). SCFAs, including butyrate, have been demonstrated to increase the production of anti-inflammatory cytokines and to influence the immune system, potentially leading to decreased MHC class II expression on enterocytes (Ji et al. 2024). This decrease in MHC expression might lessen donor T cell activation and, in turn, the severity of GVHD. Furthermore, the development and activity of Tregs, which are essential for preserving immunological tolerance, might be influenced by the gut microbiota. Treg proliferation can be encouraged by a healthy gut microbiota, which can inhibit the activation of effector T cells that cause GVHD (Lee et al. 2024). Conversely, dysbiosis, or an imbalance in the gut microbiota, can exacerbate GVHD by causing a decline in Treg populations and an increase in pro-inflammatory reactions. Probiotic usage or dietary changes that target the gut microbiota may improve MHC modulation and the prognosis of patients undergoing HCT. Restoring a healthy microbiome, for example, might lower the probability of aGVHD initiation by downregulating MHC class II expression on enterocytes.

## Effects of allogeneic stem cell transplantation on the microbiota

Observations have shown that HSCT leads to notable alterations in the composition of microbiota, affecting both its diversity and taxonomy (Chong and Koh 2020). These changes may have an impact on outcomes associated with transplantation, including overall survival (OS), infectious complications, GVHD incidence, and transplant-related mortality (TRM).A diminished variety of intestinalmicroorganisms after HSCT has been associated with inferior survival (Jenq et al. 2015). Specifically, a lack of variety in microorganisms during engraftment is strongly linked to an increased risk of TRM and a considerable decrease in overall survival (Gu et al. 2022; Taur et al. 2014).

Samples from patients with lower microbial diversity tended to contain more of the following genera: *Lactobacillus*, *Enterobacteriaceae*, *Streptococcus*, and *Enterococcus* (Taur et al. 2014). Notably, proteobacteria, particularly Enterobacteriaceae, were abundant in the blood of non-survivorpatients, whereas Actinomycetaceae and Lachnospiraceae were more prevalent in the blood of survivors.

### The gut microbiota and antibiotics

The gut microbiota composition of candidates for HSCT can be significantly altered by multiple courses of broad-spectrum antibiotics (Shallis et al. 2018). The administration of antimicrobial regimens, both prior to and following transplantation, has a profound impact on the microbiota, leading to notable consequences. Different classes of antibiotics affect the gut microbiota based on their spectrum of activity, resulting in specific pro-inflammatory patterns within the gut flora. Broad-spectrum antibiotics tend to reduce both the diversity and abundance of microbiota. Broad-spectrum antibiotics decrease the variety and abundance of microbiota. In studies involving mice, treatment with levofloxacin and cefepime resulted in an increased population of Clostridia bacteria compared to those treated with meropenem (Hayase et al. 2022). This difference was reflected in the levels of butyrate found in fecal samples. Additionally, mice treated with levofloxacin and cefepime showed significantly lower levels of Bacteroides thetaiotaomicron compared to those receiving meropenem, which has been associated with the growth of this gram-negative bacterium. Bacteroides thetaiotaomicron thrives in anaerobic environments and is capable of breaking down complex carbohydrates found in food and substances like mucin. Clostridia contribute to intestinal immunity by producing short-chain fatty acids (SCFAs), while Bacteroides thetaiotaomicron plays a role in maintaining mucus integrity. This relationship may help explain the connection between carbapenem use, often prescribed for febrile neutropenia before engraftment, and the increased severity of GVH (Hayase et al. 2022).

Antibiotics used in the context of HSCT may influence mortality associated with GVHD by altering the microbiota composition (Peled et al. 2020a). Among the 12 commonly used antibiotics, certain studies have identified piperacillin-tazobactam and imipenem-cilastatin as being linked to distinct mortality rates related to GVHD in patients with T-cell-replete HSCT. The use of these antibiotics is associated with a higher incidence of grade II-IV acute graft-versus-host disease (aGVHD), particularly affecting the upper gastrointestinal tract. Conversely, the presence of aztreonam or cefepime has been associated with lower GVHD-related mortality. Intestinal Clostridiales are known to regulate anti-inflammatory processes by activating regulatory T cells (Tregs) through SCFA metabolites, and imipenem-cilastatin treatment has been shown to reduce their abundance in both human and mouse microbiomes (Shono et al. 2016). The overgrowth of enterococci can occur due to the reduction of commensal bacteria caused by broad-spectrum systemic antibiotics and antibiotic prophylaxis, such as ciprofloxacin (Holler et al. 2014).

In addition to treating infections, antibiotic therapy serves as a method for gut decontamination. One study compared two decontamination regimens: one group of patients received 500 mg of ciprofloxacin twice daily and 500 mg of metronidazole three times daily from eight days before HSCT to 14 days after engraftment, while another group received 200 mg of rifaximin twice daily. The results indicated that gut decontamination with rifaximin had similar rates of infectious complications compared to ciprofloxacin and metronidazole. However, rifaximin also preserved a higher diversity of intestinal microbiota and mitigated the adverse effects of systemic antibiotics on microbial composition (Weber et al. 2016). Patients with prior hospitalizations showed a decrease in the expression of dominant bacterial strains that coexist harmoniously with the body, along with an increase in enterococci compared to first-time admitted patient (Spadea et al. 2022). To minimize the impact of intestinal GVHD, it is advisable to select antibiotics with a narrow spectrum of activity, particularly those that specifically target anaerobic bacteria, thereby reducing alterations in the microbiota (Lee et al. 2019). This helps minimize changes in the microbiota, as shown in Table 1.

**Table 1:** Conditioning regimen-related changes in microbiota

Population	Microbiota Changes	Antibiotic	Effect	References
Mice	↓*Bacteroides thetaiotaomicron* ↑*Enterococcus* ↑*Clostridiales*	Cefepime and Levofloxacin vs. meropenem	↓Severe GVHD	Hayase et al. (2022)
T-cell-replete HSCT human and mice	↓*Actinobacteria* ↓*Clostridiales* ↑*Erisipelotrichia* *Enterococcus* ↔ *Clostridiales*	Piperacillintazobactam Imipenem-cilastatinAzteronam and Cefepime	↑Grade II-IVaGVHD ↑GVHD-related mortality ↓GVHD-related mortality	Shono et al. (2016)
Human HSCT	Ciprofloxacin and metronidazole vs. rifaximin	↔ *Enterobacteriaceae*	↓GI-GVHD ↓TRM ↑OS	Weber et al. (2016)
Human HSCT	↑*Enterococci* classical commensal bacteria	Ciprofloxacin and broad-spectrum antibiotics	↑GI-GVHD	Holler et al. (2014)

### Impact of conditioning on the gut microbiome

By the time patients reach the transplantation stage, their gut microbiota is often significantly altered due to multiple exposures to antibiotics and chemotherapy treatments (Montassier et al. 2014). This exposure typically leads to a compromised microbiota, characterized by a reduction in α-diversity. Additionally, the conditioning regimen, which may include radiotherapy, can further diminish the microbiota. While the effects of chemotherapy on intestinal microbiota are well established, the specific mechanisms behind these effects remain incompletely understood (DeFilipp et al. 2019). Research findings regarding changes in the composition of the microbiota after HSCT have been inconsistent. For instance, studies have shown that patients exhibit higher levels of enterococci and lower levels of Firmicutes and other bacterial species before and after HSCT (Chiusolo et al. 2015; Stein-Thoeringer et al. 2019). In a recent study comparing HSCT patients to healthy controls, it was found that Actinobacteria were more prevalent in the control group, while the HSCT group had a higher prevalence of Proteobacteria and Verrucomicrobia. At the genus level, HSCT patients showed a lower abundance of *Faecalibacterium*, *Alistipes*, and *Prevotella*, but an increase in *Bacteroides*, *Klebsiella*, *Escherichia*/*Shigella*, and *Akkermansi* (Kouidhi et al. 2021). Radiation-induced enteritis can be exacerbated by conditioning regimens that involve radiation, as these regimens are known to cause dysbiosis in the microbiome. Extensive experience in oncology, particularly with the use of total body irradiation (TBI), supports this observation, highlighting the microbial loss associated with such treatments (Crawford and Gordon 2005).

### Microbiota and diet

Nutrition and diet play an often underestimated yet increasingly significant role in the health of patients undergoing HSCT. There is growing evidence linking the gut microbiota, clinical outcomes, and dietary habits (Jian et al. 2021; Shono and van den Brink 2018). Although the precise mechanisms are not fully understood, some studies indicate that diet may influence the development of beneficial microbiota in HSCT patients. Research suggests that enteral nutrition, as opposed to parenteral nutrition, can help prevent GVHD, reduce treatment-related mortality (TRM), and improve overall survival (Beckerson et al. 2019; Beckman et al. 2021).

In a study, interesting discoveries were made regarding the detrimental effects of obesity on the outcomes of HSCT in both mice and humans (Muratore et al. 2022). Obesity is associated with an increased risk of gastrointestinal involvement in a-GVHD (Khuat et al. 2020). This phenomenon is attributed to donor CD4 + T cells, which produce higher levels of pro-inflammatory cytokines that specifically affect the digestive tract. Animal studies indicate that the microbiota established by a person's diet prior to transplantation can influence TRM, often linked to adverse GVHD in the gastrointestinal system. Diet-induced obesity (DIO) in mice is associated with decreased gut microbial diversity, increased gut permeability, and enhanced endotoxin transport across the gut barrier. Following HSCT, these changes in DIO mice worsened the clinical symptoms of acute GVHD (Gleimer et al. 2015).

## Physiological function of GM

The human body is influenced by trillions of microorganisms that significantly affect various aspects of health, including immunological control (Altveş et al. 2020). Intestinal symbiotic components that develop early in childhood can protect the host from gastrointestinal infections through many processes, including pathogen inhibition. Next-generation sequencing (NGS) has dramatically enhanced our understanding of the correlation between gut bacteria and the likelihood of developing diseases (Zhang et al. 2021). In the last ten years, gut bacteria have been recognized as crucial regulators of allogeneic responses and the lethality associated with GVHD following allo-HSCT.

### GM and host physiology

Interactions between the host and gut bacteria primarily occur on the mucosal surface. It is well established that microbial communities in different areas of the human body have distinct compositions. The human intestinal mucosa covers a surface area of 250 to 400 square meters and contains between 10^13^ and 10^14^ bacteria. The intestinal epithelium is composed of a single layer of cells known as IECs, which include Paneth cells, goblet cells, and intestinal stem cells (Riwes and Reddy 2020). IECs serve two main functions: (1) they act as a barrier that separates the intestinal canal from its contents and (2) facilitate nutrient absorption. Beneficial gut symbiotic microbes can aid in restoring the intestinal epithelial barrier, protecting the host from pathogens (Barbara et al. 2021). Beneficial gut symbiotic microbes can enhance the restoration of the intestinal epithelial barrier by shielding the host against pathogens.

Paneth and goblet cells are crucial in preventing bacteria from penetrating host tissues. They play an essential role in maintaining a dynamic balance between the intestinal microbiota and the immune response in the small intestine (Norona et al. 2020) Paneth cells, primarily located in the crypts of the small intestine, produce antimicrobial compounds such as α-defensins, which are vital for regulating bacterial populations in the intestine (Ara et al. 2020). A reduction in Paneth cells during GVHD leads to decreased levels of α-defensins, resulting in diminished diversity within the gut microbial community and changes in its composition (Araujo 2020). Goblet cells secrete mucin, creating a protective layer in the intestinal lumen. Damage to goblet cells in individuals with GVHD can lead to the migration and spread of harmful luminal pathogens, worsening GVHD and increasing the risk of infection (Teshima et al. 2016). Paneth cells are crucial for controlling bacterial population density in the small intestine. There is a correlation between the number of Pane's cells and the therapeutic response to GVHD (Hou et al. 2021). Additionally, the number of Pane cells is inversely related to the severity and prognosis of clinical GVHD. This serves as a histopathological grade index for G-GVHD. Research has shown that a group of pattern recognition receptors (PRRs) in IECs can activate Paneth cells to produce and release significant amounts of α-defensins stored in cytoplasmic granule (Delgado Betancourt 2024). This process does not require the detection of microbe-associated molecular patterns (MAMPs). The production of REG-IIIγ is dependent on signals from the microbiota. Under normal conditions, the movement of bacteria or bacterial components through the intestinal barrier is regulated by how MAMPs interact with the host's immune cells involved in inflammation. Inflammatory diseases, such as inflammatory bowel disease, are characterized by a decrease in Paneth cells and changes in the intestinal microbiota. This suggests that enhancing the secretion of antimicrobial peptides by Paneth cells could be beneficial in preventing the onset of GVHD.

### GM and body immunity

The gut microbiota plays a vital role in regulating both local and systemic immune responses. However, the exact mechanisms by which it coordinates immune reactions and modulates immunological responses following allo-HSCT are not yet fully understood (Fiorenza and Turtle 2021). Researchers have explored the relationship between immune system recovery, clinical outcomes, and intestinal microbes in children undergoing allo-HSCT (Ingham et al. 2019). Their findings indicated that patients with higher levels of Ruminococcaceae and Trichospirococcaceae experienced more effective reestablishment of natural killer (NK) and B cells, with only mild or no acute GVHD observed, along with improved overall survival. Studies have shown that certain beneficial bacteria can initiate the effector T-cell response, influencing the treatment of hematologic malignancies in patients undergoing allo-HSCT. For instance, patients with a greater abundance of Eubacterium myxus exhibited a lower risk of recurrence and slower disease progression. One study demonstrated that a combination of 11 bacterial strains significantly enhanced the immune response of IFN-γ + CD8 + T cells in mice, leading to a stronger anti-tumor immune response against *Eubacterium myxus* (Vicente-Dueñas et al. 2020). This resulted in an improved anti-tumor immune response against Eubacteriummyxus. Intestinal bacteria also affect CD4 + T helper cells. A study demonstrated that the colonization of mouse intestines by filamentous bacteria could stimulate the differentiation of primitive CD4 + T cells into cells that are capable of generating IL17 and IL22(van Lier et al. 2020). IL22 is a cytokine produced by T helper cells, specifically Th17 cells. In healthy human volunteers, circulating memory CD4 + T lymphocytes have been observed to release Th17 and Th1 cytokines in response to gut microbes (Ingham et al. 2021). The balance between Th17 cells and regulatory T cells (Tregs) is crucial for the development of GVHD in inflammatory disorders. Additionally, IL-6 is a key inflammatory mediator that inhibits Th17 development, disrupting the balance between Th17 and Treg cells. Inhibiting the IL-6 signaling pathway with IL-6 antibodies can significantly reduce the severity of GVHD.

### Effect of GM on graft-versus-leukemia (GVL)

The primary goal of allo-HSCT is to restore the immune system in patients with malignant hematologic disorders while eliminating any remaining tumor cells, thereby reducing the risk of recurrence (Andrlová et al. 2021). This effect is referred to as the graft-versus-leukemia (GVL) effect. Research has shown that genetically modified (GM) substances and their byproducts can modulate the immune response between donor and recipient cells, leading to a decrease in the incidence of GVHD and an enhancement of the GVL effect (Deeg 2021). GM can control the characteristics of mucosal-associated invariant T (MAIT), γδT, and invariant natural killer T (iNKT) cells. Natural killer T (NKT) cells and other non-classical T cells enhance the body's immunological reconstitution process, enhancing the GVL effect. GM can influence the characteristics of mucosal-associated invariant T (MAIT) cells, γδ T cells, and invariant natural killer T (iNKT) cells. Natural killer T (NKT) cells and other non-classical T cells play a significant role in enhancing the body's immunological reconstitution, thereby improving the GVL effect. According to research, the secretion of glucagon-like peptide-2 (GLP-2) by intestinal stem cells and Paneth cells was found to be reduced in mice with acute GVHD and in patients who underwent allo-HSCT. Administering the GLP-2 analog Teduglutide externally was shown to decrease the frequency of severe GVHD in patients with acute GVHD who were resistant to corticosteroids, without negatively affecting the GVL effect (Norona et al. 2020).

## Graft-versus-host disease mechanisms

**Fig. 3:**
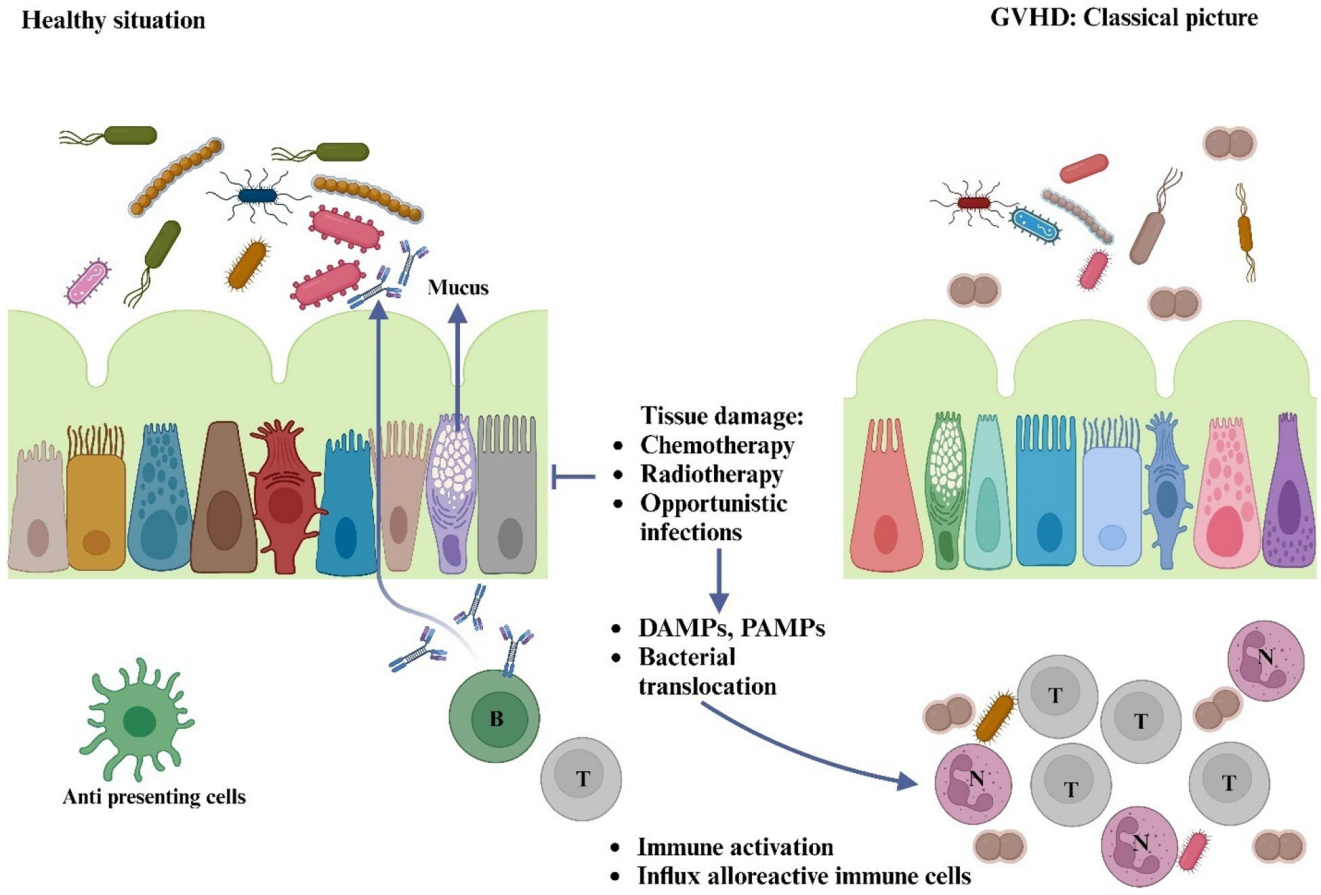
Shows that in GVHD, chemotherapy/radiotherapy damages tissue and translocates microorganisms, generating DAMP and PAMP that activate host APCs. APCs excite donor lymphocytes, which produce cytokines and recruit effector cells, damaging and inflaming tissue

There are two main types of graft-versus-host disease (GVHD). The first type, acute GVHD, occurs within 100 days following allogeneic hematopoietic cell transplantation (allo-HCT). The second type, which can be classified as late-onset acute or chronic GVHD, develops after 100 days post-transplant. Symptoms of acute GVHD include a rash, jaundice, nausea, abdominal cramps, diarrhea, and a reduced ability of the immune system to combat infection (Zeiser and Blazar 2017a). Chronic GVHD is primarily characterized by sclerosing disease, which can affect nearly any organ in the body. Patients undergoing allo-HCT typically receive preventive immunosuppressive therapy, which may include medications such as methotrexate, calcineurin inhibitors, post-transplantation cyclophosphamide, anti-thymocyte globulin (ATG), and mycophenolic acid, often in combination, to help prevent GVHD (Sharma 2023). Despite these preventive measures, approximately 30–50% of patients still experience acute GVHD. Alloreactivity and tissue damage are recognized as key factors in the traditional understanding of acute GVHD pathogenesis. Conditioning therapies, including radiotherapy and chemotherapy, can harm the intestinal mucosa and induce inflammation (mucositis), which compromises the intestinal barrier and increases the risk of bacterial translocation, as shown in Fig. 3 (Jenq and Van den Brink 2010; Zeiser and Blazar 2017b). Damage to the body causes the release of molecules known as pathogen- and damage-associated molecular patterns (DAMPs and PAMPs), which in turn stimulate both hematopoietic and non-hematopoietic antigen-presenting cells (APCs). Donor immune cells are primed and differentiated more effectively by these APCs, which also release inflammatory cytokines. Tissue injury and inflammation are exacerbated when effector cells and activated neutrophils are brought into the tissues, where they release additional inflammatory cytokines. Similar mechanisms are thought to be involved in the development of both the curative graft-versus-leukemia (GVL) response and chronic GVHD (Fowler 2006). Within the pathophysiology of acute GVHD, DAMPs such as ATP, uric acid, and IL-33 play a crucial role. How these DAMPs contribute to the onset and severity of GVHD has been investigated. The release of ATP from injured cells serves as a DAMP that triggers immunological reactions. It has been shown that purinergic receptors, particularly P2X7 and P2Y2, which are implicated in the inflammatory response during GVHD, can be activated by ATP. The severity of GVHD is increased when these receptors are activated because it causes the release of pro-inflammatory cytokines and the recruitment of immune cells. In both human and mouse models of GVHD, elevated ATP levels in the peritoneal fluid have been noted, suggesting that it may be a promising target for therapy (Wilhelm et al. 2010). Another important DAMP that has been investigated in connection with GVHD is uric acid (UA). Increased inflammation and tissue damage during allo-HCT have been linked to elevated uric acid levels. Studies have indicated that uric acid can trigger the NLRP3 inflammasome, resulting in the generation of inflammatory cytokines that aid in the pathophysiology of GVHD. Given its complex involvement in GVHD, some research indicates that low uric acid levels may be associated with an increased risk of developing the condition (Ghasemi et al. 2020). Another study demonstrate that intestine commensal bacteria and the damage-associated molecular pattern uric acid contribute to the generation of IL-1 mediated by the Nlrp3 inflammasome during conditioning therapy, and that gastrointestinal cleaning and uric acid depletion decreased the severity of GvHD. Survival was enhanced by early IL-1 inhibition or genetic deficiencies in the IL-1 receptor in T cells and dendritic cells (DCs). The complete manifestation of GvHD depended on the Nlrp3 inflammasome components Nlrp3 and Asc, which are necessary for pro-IL-1 cleavage (Jankovic et al. 2013). Modification of uric acid levels has also been investigated as a possible tactic to lessen the severity of GVHD. Another cytokine that has been shown to exacerbate the inflammatory response in GVHD is IL-33, which increases tissue damage and the disease's severity. According to studies, by modifying the immune response, IL-33 signaling pathway targeting may offer a treatment option to lessen the severity of GVHD (Kobayashi et al. 2017).

### Evidence for microbiota's role in GVHD, transplantation issues

It is crucial to have a wide range of anaerobic commensal bacteria in steady-state conditions. These bacteria play a vital role in preventing the growth of harmful pathogens, producing important nutrients, and keeping the intestinal system balanced by interacting with the mucosal immune system (van Lier et al. 2023). Through the breakdown of dietary polysaccharides into short-chain fatty acids (SCFAs) like butyrate, propionate, and acetate, the gut microbiota plays a crucial role in supporting a tolerant intestinal mucosal immune system. These SCFAs possess immunomodulatory properties, contributing to the overall balance of the immune system (Rooks and Garrett 2016). By inhibiting histone deacetylation, SCFAs encourage Tregs to produce the anti-inflammatory IL-10 and innate lymphoid cells (ILCs) to produce IL-22, which improves epithelial integrity (Smith et al. 2013; Sun et al. 2018; Yang et al. 2020). Additionally, B lymphocytes secrete immunoglobulin A (IgA) in response to SCFAs, providing an initial line of defense against pathogens that have infiltrated the body (Kim et al. 2016).In addition, SCFAs and other bacterial metabolites, such as polyamines, enhance gut barrier function by encouraging Goblet cells to generate mucus and increasing the synthesis of tight junction proteins in endothelial cells (Gaudier et al. 2004).

The compositional and functional arrangement of microbial communities in the human gut may now be quickly and extensively explored because of developments in sequencing technology. As a result, several research has been conducted to investigate the connection between microbiota and human health (Le 2003). These studies have shown that harm to the bacteria in the intestines is linked to many immune-related disorders, such as GVHD (Ruff et al. 2020). In 2012, it was documented that there is a decrease in microbial diversity soon after allogeneic HCT, and this decrease occurs before the onset of GVHD (Jenq et al. 2012a; Taur et al. 2012). A subsequent, more extensive investigation revealed that patients who preserved a diverse microbiota had a considerably reduced likelihood of death caused by GVHD or opportunistic infections, resulting in improved overall survival compared to patients who suffered a decline in microbiome diversity (Taur et al. 2014). A recent international multicenter investigation, which analyzed over 8000 samples from 1362 distinct patients, has proven the link between limited microbial diversity and a higher risk of mortality associated with transplantation and GVHD (Peled et al. 2020). Curiously, this connection needed to be clarified in patients who received a lymphocyte-depleted allograft. This implies that the presence of donor-derived adaptive immune cells that react to foreign tissue plays a significant role in the mortality linked to disturbance of the microbiota. In a mouse model of allogeneic HCT, it was demonstrated that radiotherapy-induced bacterial translocation results in the attraction of neutrophils and, by activating alloreactive T lymphocytes, leads to the development of acute GVHD (Hülsdünker et al. 2018).

Decreased diversity of the microbiota may contribute to other issues associated with transplantation that were previously seen but not fully comprehended. Obesity is linked to negative consequences following allogeneic HCT. A study using a mouse HCT model showed that obesity, combined with a reduced variety of microbiota, results in an inflammatory gut environment and a higher likelihood of GVHD. A lifetime elevated risk of cardiovascular events and subsequent malignancies awaits allogeneic HCT recipients who make it through the first 1–2 years after transplantation. Decreased diversity of the microbiome is associated with an increased likelihood of experiencing cardiovascular events, cancers, and overall poor health (Schroeder and Bäckhed 2016).

The microbiota of allogeneic HCT survivors who went on to acquire secondary malignancies was shown to be more disturbed in cross-sectional research than in long-term survivors who did not experience secondary malignancies (Hino et al. 2023). The long-term difficulties in survivors of allogeneic hematopoietic cell transplantation (HCT) may be connected to damage to the microbiome that occurs shortly after the transplantation. However, there is currently a lack of definitive evidence to support this claim.

### Disruptive factors of the microbiome

**Fig. 4:**
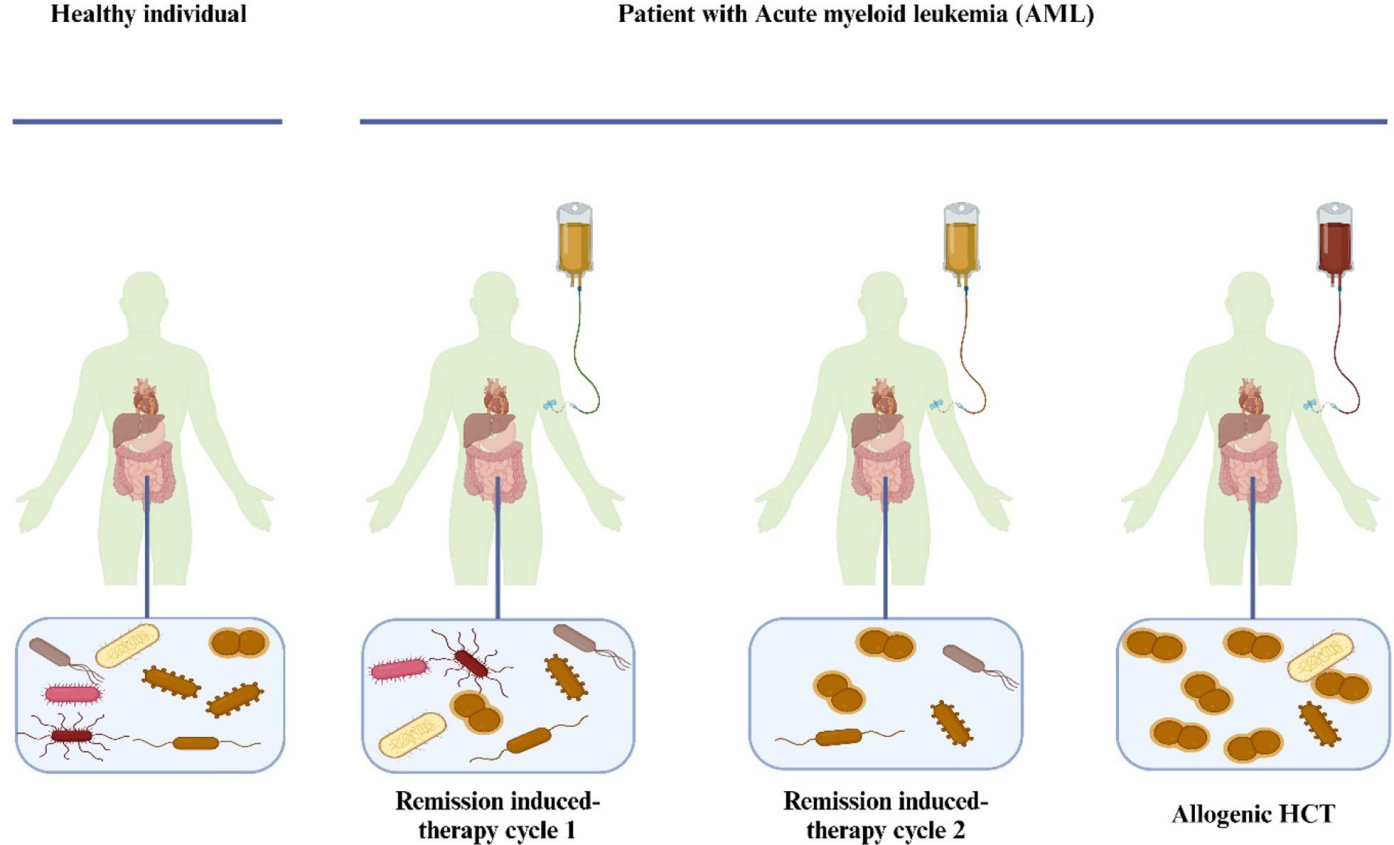
A typical treatment plan for acute myeloid leukemia (AML) is shown, consisting of two cycles of chemotherapy to induce AML remission, followed by allogeneic stem cell transplantation. Repeated cycles of remission-induction chemotherapy and subsequent allogeneic HCT are associated with alterations in diet and necessitate the use of antibiotics, anti-emetics, and other drugs that lead to loss of microbiome diversity. In addition, chemotherapy and radiotherapy directly damage the microbiome

Before transplantation, during the remission-induced chemotherapy period, and in the initial post-transplantation days, there is a drop in intestinal microbial diversity Fig. 4 (Rashidi et al. 2020, 2022; Taur et al. 2012). When compared to the intestinal microbiome of healthy people, the post-HCT microbiome typically exhibits reduced diversity, decreased commensal abundance, and a high frequency of colonization by *Enterococcus*, *Streptococcus*, or Proteobacteria (Peled et al. 2020). During allogeneic HCT, several factors have been connected to early microbiome damage. Typically, recipients of allogeneic hematopoietic stem cells (HCT) undergo several rounds of chemotherapy, occasionally in conjunction with total body irradiation (TBI), to minimize tumor burden and suppress the recipients' adaptive immune system, therefore preventing transplant rejection. Loss of microbial diversity and changes in the composition of the microbiome have been linked to the toxicity of both treatments (Montassier et al. 2015; Shouval et al. 2023). Both direct and indirect effects on gut bacteria are possible from this toxicity, such as the death of Paneth cells. Paneth cells are secretory cells that generate antimicrobial substances to maintain host-microbial balance, and they are found near the base of the intestinal crypt (Cray et al. 2021). Intestinal Paneth cells are less prevalent in patients with acute GvHD, however in a GvHD animal model, Paneth cells treated with glucagon-like peptide-2 reversed microbiome damage (Norona et al. 2020). The effect of antibiotics on the microbiome, which is commonly thought to be the source of microbiome damage in allogeneic HCT recipients, has been investigated the most to date. In allogeneic HCT recipients, antibiotic exposure is both high and prolonged. Bacterial translocation is more likely in patients with chemotherapy-induced mucositis of the gastrointestinal tract and mouth (Tamburini et al. 2018), while neutropenia is commonly associated with conditioning chemotherapy regimens and remission-induction chemotherapy regimens. Empiric broad-spectrum antibiotics are consequently frequently administered to patients during episodes of neutropenic fever as prophylactic measures to prevent systemic infections. According to retrospective research, the early use of broad-spectrum antibiotics (between day − 7 and day 0 before allogeneic HCT) reduced the quantity of *Clostridiales* and was linked to increased transplant-related mortality (Weber et al. 2017). Moreover, the administration of specific antibiotics was linked to an increased probability of intestine colonization and consequent bloodstream infections by the corresponding dominant species (Taur et al. 2012; Ubeda et al. 2010). Nonetheless, other medications can also adversely affect the variety of the microbiome, not just antibiotics. In two sizable observational studies conducted in the healthy population of The Netherlands and Belgium, it was shown that nearly any medication could disrupt the microbiota (Falony et al. 2016; Zhernakova et al. 2016). Antiemetics, proton pump inhibitors, antidepressants, and opiates are among the commonly used medications that have been shown to alter the microbiome's composition in a community setting. As a result, these medications may have an effect on the microbiota of allogeneic HCT patients.Prolonged immunodeficiency, a feature of allogeneic HCT, could be another significant factor disrupting intestinal homeostasis. Microbes that a newborn comes into contact with from birth and their interactions with their developing immune system shape their microbiome (Dominguez-Bello et al. 2010; Ley et al. 2006). This mutual interaction between the immune system and the microbiota is put under a lot of strain following allogeneic HCT since it usually takes months to years for the donor's adaptive immune system to reconstitute after transplant. This could cause the microbiome's recovery to be markedly delayed. Immunosuppressive medication use was the single most significant factor in solid organ transplant recipients' microbiome damage, which persisted for years following transplantation (Swarte et al. 2022). The microbiome has been shown to influence T cell recovery in particular and the recovery of innate and adaptive immunity in general in allogeneic HCT, confirming the mutual relationship between the immune system and the microbiota (Miltiadous et al. 2022; Schluter et al. 2020).

**Fig. 5:**
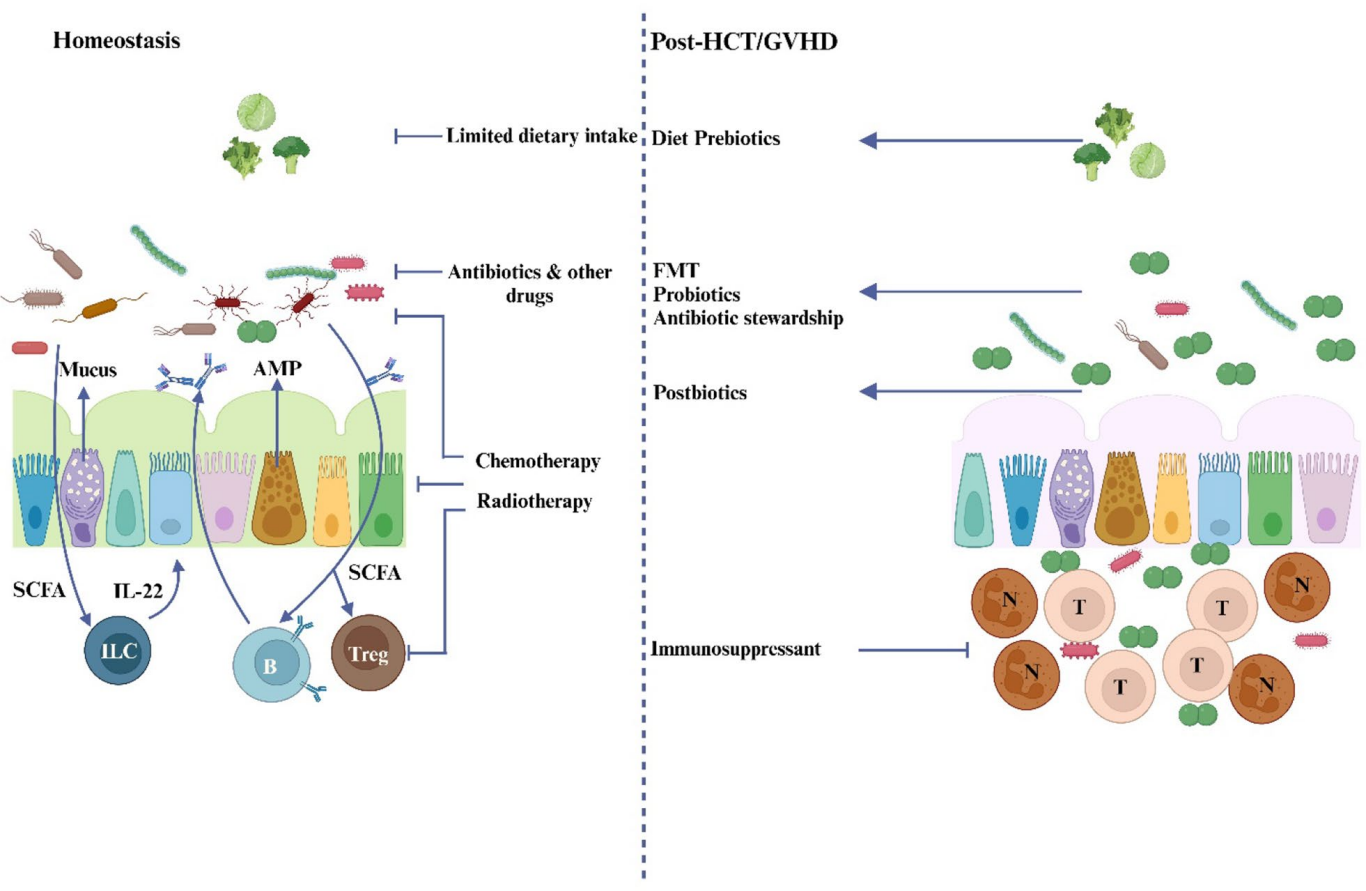
Gut homeostasis disruption and restoration in GVHD. In a healthy state, commensals break down dietary fibers into short-chain fatty acids (SCFA) (left panel). These SCFA stimulate the development of regulatory T cells (Treg) and cause B cells to secrete IgA (b). Through innate lymphoid cells (ILC), SFCA increase the production of IL-22, maintaining epithelial integrity and encouraging the creation of anti-microbial peptides (AMPs), which are crucial for regulating the microbial community and preventing pathogen expansion. To prevent bacterial translocation, goblet cells secrete mucus. The use of antibiotics, dietary modifications, and other aspects of cancer treatment all have an effect on all of these processes. This leads to dysbiosis, low SCFA levels, impaired mucus production, injury to epithelial cells, activation and influx of (alloreactive) T lymphocytes (t) and neutrophils (n), and inflammation are the outcomes (right panel). While immunosuppressive medications are the mainstay of traditional GVHD treatment, other cutting-edge strategies include microbiota-targeted treatments that either prevent or treat dysbiosis

Finally, a person's diet greatly affects the components of their gut microbiota (Johnson et al. 2019; Kolodziejczyk et al. 2019). Because of the pain, nausea, and decreased appetite brought on by mucositis, allogeneic HCT recipients often have reduced oral intake. In order to guarantee adequate caloric intake, parenteral feeding is needed (Kyle et al. 2005). Parenteral feeding was found to be less effective than enteral nutrition in maintaining the diversity and composition of the microbiome in two small cohorts (Andersen et al. 2020; D’Amico et al. 2019). In addition, two retrospective investigations revealed that patients receiving parenteral nourishment had worse survival rates and a higher prevalence of gastro-intestinal GVHD in comparison to those getting appropriate enteral nutrition (Beckerson et al. 2019; Gonzales et al. 2018). Together, these elements have the potential to significantly change the population of gut microbiota and interfere with intestinal homeostasis. The knowledge gained from the several investigations previously stated resulted in a revised model of GVHD pathophysiology that included the significance of the gut microbiota as a regulator of immunologic tolerance and a custodian of gut homeostasis Fig. 5 (Shono and van den Brink 2018).

## GM and GVHD

It has long been believed that the gut microbiota and the onset of acute GVHD are related. Using murine allo-SCT models, it was shown in the 1970s that eliminating the gut microbiota stops acute GVHD from developing in germ-free (bacteria-free) mice, which have less severe acute GVHD than ordinary animals (Bekkum et al. 1974). The prophylactic impact of eliminating gut microbiota with oral non-absorbable antibiotics (also known as “gut decontamination”) against acute GVHD was then assessed in a number of clinical trials; however, no discernible benefit was found (Passweg et al. 1998; Vossen et al. 1990). A more recent retrospective analysis, however, revealed that although the overall incidence of acute GVHD was relatively low (8%), pediatric patients who underwent effective whole gut cleansing had a considerably lower rate of acute GVHD. In this investigation, stool cultures that tested negative for both bacteria and fungi in at least three of five samples taken between days − 10 and + 30 were deemed to have successfully decontaminated the entire gut (Vossen et al. 2014). After SCT, GVHD mice in a mouse model show a decrease in bacterial diversity (Jenq et al. 2012b). Mice given ampicillin prior to SCT (days − 21 to − 14) after developing GVHD exhibit a decrease of Blautia and the appearance of *Enterococcus*. In contrast to ampicillin-treated mice lacking *Lactobacillus johnsonii*, mice treated with *Lactobacillus johnsonii* (day − 12 to − 2) after ampicillin displayed dominant *L. johnsonii*, no intestinal tract expansion of Enterococcus, and less severe GVHD (Jenq et al. 2012b). In recent years, human studies have also examined the relationship between the gut microbiota and acute GVHD and/or transplant outcomes (Table 1). GVHD patients show a reduction of intestinal variety, which is comparable to the mice model. Additionally, GVHD patients exhibit more *Lactobacillales* and less Clostridiales, but non-GVHD patients do not exhibit similar microbial alterations (Jenq et al. 2012b). Intestinal microorganisms consist of several microbial constituents, including bacteria, fungi, and viruses, and the interactions between these constituents are complex. Maintaining homeostasis and regulating metabolites are crucial for immunity and overall health. GVHD, an immunological illness, is the primary consequence of allo-HSCT and significantly affects the prognosis of patients undergoing allo-HSCT (Mohamed et al. 2021). The relationship between GM diversity and metabolites and the presence and progression of allo-HSCT-related GVHD was significant (Masetti et al. 2023). Bile acids can alter intestinal health and immunological responses, which can affect the severity of aGVHD, according to recent research. One of the two main ways that tauroursodeoxycholic acid (TUDCA) has been demonstrated to lessen the severity of aGVHD is by improving cell viability in the presence of pro-inflammatory cytokines and decreasing intestinal antigen presentation. This decrease in intestinal epithelial cell death results from this reduction in antigen presentation, and this is essential for reducing the inflammatory response linked to aGVHD (Haring et al. 2020). Additionally, GVHD has been inked to exacerbated T cell-driven inflammation due to altered bile acid metabolism. It has been shown that alterations in the gut microbiota might result in abnormalities in the metabolism of bile acids, which can exacerbate immunological reactions and make aGVHD more severe (Lindner et al. 2024). Numerous theories have been put out to explain how the gut microbiota affects the progression of GVHD **(**Table 2**).**

**Table 2:** Mechanisms via which the gut microbiome could affect GVHD

Mechanism	Host involvement	Role of microbiota	References
Bacterial translocation–associated inflammation	Pattern recognition receptors detect bacterial MAMPs and recruit inflammatory cells	Bacteria and their associated MAMPs translocate across the disrupted gastrointestinal barrier	Cooke et al. (2001), Gerbitz et al. (2004), Schwab et al. (2014)
Altered balance of Tregs versus Th1/Th17 cells	Reduced butyrate levels affect signaling via inflammatory pathways and prevent Tregs from homing to the gut mucosa		Arpaia et al. (2013), Atarashi et al. (2013), Furusawa et al. (2013)
Decreased HDAC inhibitor activity	IEC and Treg function are both improved by HDAC inhibition		Gilbert et al. (2003, 2005), Furusawa et al. (2013)
Impaired intestinal barrier integrity	Limited butyrate reduces barrier integrity by impeding IEC repair and wound healing	Reduced Clostridiales abundance, or dysbiosis, lowers the generation of SCFAs, such as butyrate, which is an essential energy source for IECs and triggers Tregs and HDAC inhibition	Ma et al. (2012), Mathewson et al. (2016), Vinolo et al. (2011)
Loss of mucus barrier	Treatment with imipenem-cilastatin in GvHD-affected mice is linked to elevated concentrations of the mucinolytic bacterium Akkermansiamuciniphila, mucus layer thinning, and impaired intestinal barrier function	Could provide more chances for bacteria and MAMPs to activate immune cells	Ara et al. (2018), Shono et al. (2016)
Reduced antimicrobial peptide (AMP) production	The small intestine's Paneth cells, which generate AMPs, are a sign of increased risk for GvHD. Paneth cells produce α-defensins, or AMPs, which block GvHD-mediated dysbiosis	Prevents GvHD-mediated dysbiosis in mice by boosting AMP (α-defensin) synthesis and preserving the variety and balance of the gut microbiota	Eriguchi et al. (2015), Levine et al., (2013)
IL-17	HCT recipient mouse-derived IL-17A protects against acute GvHD	Increased vulnerability to acute GvHD is transferred to WT mice when the intestinal microbiota of IL-17 receptor-KO mice is transferred	Varelias et al. (2017)
Neutrophils	Neutrophils in human GvHD lesions are correlated with GvHD severity. In mice, GvHD-related mortality is decreased by neutrophil depletion. In order to transmit alloantigen to donor T cells, recipient neutrophils in mice transport it from the intestinal mucosa to lymph nodes	Microbiota-dependent recruitment occurs in the intestines	Hülsdünker et al. (2018), Schwab et al. (2014)
Tryptophan	IL-22 is produced by activated ILCs (see IL-22 below)	ILCs are activated by tryptophan metabolites produced by lactobacilli	Zelante et al. (2013)
IL-22	Encourages intestinal stem cell-mediated epithelium regeneration and is linked to a less severe case of GvHD in mice	Forms the mice's intestinal microbiota	Lindemans et al. (2015), Zenewicz et al. (2013)
MAIT cells	HCT recipient MAIT cells protect from GvHD in mice, constrain alloreactive donor T cell expansion in colon, and produce IL-17A. In humans, robust MAIT cell reconstitution is associated with reduced risk of subsequent acute GvHD; MAIT cells suppress proliferation of conventional T cells	Activated by riboflavin metabolites produced by some gut bacteria; influence gut microbiota composition in mice; reconstitution is associated with increased Blautia and Bifidobacterium longum abundance in the gut in humans	Bhattacharyya et al. (2018), Varelias et al. (2018)

### Loss of GM diversity and GVHD

There was a direct relationship between intestinal inflammation caused by GVHD and alterations observed in animal models that underwent allo-HSCT and had human intestinal flora (Nguyen et al. 2021). According to a study conducted in a mouse model of GVHD, there was an observable increase in Lactobacillus and a corresponding decrease in Clostridium (Staffas et al. 2017). Eliminating Lactobacillus before HSCT will exacerbate mouse GVHD, but reinstalling dominant Lactobacillus could effectively shield mice from GVHD infiltration. A study discovered that when Lactobacillus and symbiotic bacteria levels were decreased, probiotics administered to mice as a preventive measure helped maintain the variety of intestinal microorganisms, thus avoiding the occurrence of GVHD in both mice and GVHD patients (Sadanand et al. 2019). Research has demonstrated that maintaining a varied community of mutually beneficial bacteria helps decrease the occurrence of inflammatory bowel illness and hematocrit. Additionally, those with a lower diversity of bacteria in their gut had notably higher mortality rates. A study found that the three-year survival rate of patients who received allo-HSCT was influenced by the type of intestinal microbiota they possessed (Hong et al. 2021). Specifically, patients in the low-diversity group had a survival rate of 36%, those in the medium-diversity group had a rate of 60%, and those in the high-diversity group had a rate of 67%. As per the research conducted, it has been observed that there exists an inverse correlation between the abundance of cyanobacteria and the mortality rate associated with GVHD (Ilett et al. 2020). In other words, the overall survival rate increased as the quantity of cyanobacteria increased. Thus, the diversity of intestinal microbes during transplantation can accurately predict the death of recipients undergoing allo-HSCT (Greco et al. 2021).

When the balance of the intestines is disturbed, bacteria and their antigens can move to other parts of the body, causing host immune cells to produce particular receptors that can identify patterns associated with harmful microorganisms. This can facilitate the activation of neutrophils and antigen-presenting cells (APC), augment the capacity for T-cell stimulation, and expedite the activation of diverse immune cells (Lee et al. 2021). Not only do hematopoietic cells exhibit PRR, but epithelial and endothelial cells also express PRR. This can result in the upregulation of the MHC and the secretion of inflammatory cytokines. Consequently, this triggers an inflammatory reaction caused by immune responses from different T-cells, resulting in the infiltration of target organs, cell death, and GVHD (Koyama et al. 2019).

### Viruses and GVHD

Epstein-Barr (EBV) and cytomegalovirus (CMV) are viruses commonly colonizing the intestines.

CMV, BK, and John Cunningham viruses have been identified (Ghimire et al. 2017; Michonneau et al. 2024; Sahin et al. 2016). Chemoradiotherapy therapies for HSCT patients, particularly allo-HSCT patients before transplantation, result in significant immunosuppression, activating and increasing intestinal colonizing viruses. Research has demonstrated that immunosuppression after hematopoietic stem cell transplantation might cause reactivation of CMV, resulting in the development of GVHD and an increase in transplant-related illness and death (Massoud et al. 2022). Epstein-Barr virus, a herpes virus, can potentially induce post-transplantation lymphoproliferative disease (PTLD). This condition can have deadly outcomes but typically does not result in higher death rates. Various microorganisms can modulate enteroviruses through different mechanisms. The presence of intestinal bacteria can facilitate the reproduction and dissemination of enteroviruses belonging to many families, including norovirus, picornavirus, retroviruses, and reovirus. This, in turn, increases the likelihood of GVHD. Exogenous viral infections can have both a direct effect on allo-HSCT patients and an indirect effect on patient prognosis by triggering GVHD. Research has indicated that norovirus infection can result in increased illness and death both before and during allo-HSCT transplantation (Lin and Liu 2013). Additionally, picornaviruses are frequently responsible for the common cold in the general population. However, in individuals with weakened immune systems, such as recipients of HSCT, there is a high risk of severe infection or subsequent GVHD, leading to increased mortality attributable to transplantation (Mikulska et al. 2024).

### Fungi and GVHD

Although gut bacteria are now the main focus of research on GVHD, the significance of fungi should be recognized (Ji et al. 2024). Stable Candida fungi in the intestine are a risk factor for acute graft-versus-host disease (aGVHD) (Panagopoulou and Roilides 2024). Using fluconazole as a preventive measure can decrease the likelihood of GVHD development. Dectin-1 is an essential receptor that detects B-D-glucan in fungal cell walls, namely Candida and initiates mucosal anti-fungal CD4 + and Th17 responses (Apostolopoulos et al. 2024). The secretion of IL17 and IL22 is crucial for protecting against fungal infections in mucosal tissues (Roe 2024). C-type lectin receptors detect α-mannan, a component of the Candida cell wall. This detection triggers macrophages to generate cytokines IL6 and IL23, which in turn enhances the anti-fungal activity of Th17 cells. This response occurs when C-type lectin receptors are recognized. The presence of these fungal wall components may be associated with the development of GVHD, and it is believed that the C-type lectin-mediated Th17 response plays a role in the development of acute GVHD. Research has demonstrated that the body's ability to respond to fungi through the immune system can be categorized into two types: cellular immunity and humoral immunity. In macrophages, the activation of pattern recognition receptors, including Dectin-1, Dectin-2, mannose receptor, and TLR-2, plays a crucial role in cellular immunity by triggering the Th17 response. This activation leads to the induction of the anti-Candida defense system in both the mouse and human mucosa and the host.

### GM metabolites and GVHD

The dietary constituents of microbial metabolites affect various physiological processes, including the gastrointestinal tract, immunological balance, energy metabolism, vascular function, and neurobehavior (Dmytriv et al. 2024). Gastrointestinal microbiota-derived short-chain fatty acids (SCFAs) are crucial for the pathogenesis of many illnesses. Butyric acid, a short-chain fatty acid (SCFA), controls histone acetylation. IECs rely on these cells as their primary energy source (Liu et al. 2024). Butyric acid levels in intestinal tissues decrease following allo-HSCT. Butyric acid administered through gavage increases the acetylation of histone H4 in IECs and strengthens the connections between these cells (Riwes and Reddy 2018). As a result, apoptosis and GVHD were reduced. Seventeen Clostridium strains with substantial butyric acid production were identified and chosen (Mathewson et al. 2016). They discovered that they could directly stimulate the growth of intestinal regulatory T cells (Tregs) and speed up the release of the anti-inflammatory chemical IL10 by macrophages to control inflammation. Ultimately, it is essential for maintaining gastrointestinal homeostasis and mitigating graft-versus-host diseases (GVHD). Furthermore, Tregs also hinder GVHD progression by suppressing the expansion of alloreactive T cells and T effector cells. Indole is a compound derived from l-tryptophan through the action of tryptophanase in symbiotic bacteria. It is responsible for the typical odor observed in human feces (Wu et al. 2020). It functions as a “quorum sensing” signal to control virulence and biofilm formation, maintain the integrity of the mucosal barrier, and regulate the production of pro-inflammatory and anti-inflammatory genes in IECs. Research has indicated that allo-HSCT recipients with low levels of 3-indoxyl sulfate (3-IS) in their urine samples shortly after transplantation (specifically, ten days after transplantation) have a considerably higher risk of trans-graft-related mortality one year after the allo-HSCT procedure. The indole sulfate concentration in patients with GVHD stool samples dropped from an initial level of (42.5 ± 11.0) mmol/L before transplantation to (11.8 ± 2.8) mmol/L. Further, it decreased to (3.5 ± 3.0) mmol/L after transplantation. The choice of gastrointestinal decontamination method has an impact on the early decrease in 3-IS, the progress of antibiotic therapy, and the NOD2/CARD15 genotype receptor (Routy et al. 2017). Reduced levels of 3-IS in urine samples were linked to a decrease in the variety of bacteria present, while decreased 3-IS levels in fecal samples were associated with converting critical symbiotic bacteria and Enterococcus upon admission. The degree of this change is more noticeable in patients with active gastrointestinal GVHD who receive antibiotic prophylaxis and infection treatment while experiencing neutropenia.Furthermore, strain-specific Enterococcus PCR analysis revealed that the samples from these individuals primarily contained Enterococcus faecium alone or both Enterococcus faecium and Enterococcus faecalis. This illustrates the possible efficacy of indole generated by microbiota in preserving the integrity of mucosal tissues and protecting against inflammation. Bile acids can alter intestinal health and immunological responses, which can affect the severity of aGVHD, according to recent research. One of the two main ways that tauroursodeoxycholic acid (TUDCA) has been demonstrated to lessen the severity of aGVHD is by improving cell viability in the presence of pro-inflammatory cytokines and decreasing intestinal antigen presentation. This decrease in intestinal epithelial cell death results from this reduction in antigen presentation, and this is essential for reducing the inflammatory response linked to aGVHD (Haring et al. 2020). Additionally, GVHD has been inked to exacerbated T cell-driven inflammation due to altered bile acid metabolism. It has been shown that alterations in the gut microbiota might result in abnormalities in the metabolism of bile acids, which can exacerbate immunological reactions and make aGVHD more severe (Lindner et al. 2024).

The connection between lactic acid bacteria (LAB) present in fermented foods and their existence in the human gut microbiome has been clarified by a recent large-scale genome-wide investigation by Pasolli et al. LAB are considered probiotics because of their possible health advantages and are necessary for the creation of many fermented foods. Both newly sequenced and publically available metagenomic data from food sources and human gut samples were thoroughly compared in this investigation. According to the results, both habitats contain closely related LAB strains, suggesting that eating fermented foods can increase the variety of LAB in the gut microbiome. This implies that LAB derived from food can colonize the gut, possibly improving gut health and affecting immunological responses. Additionally, the study indicated that the genomic variety of LAB strains may influence how well they function and adapt to the intestinal environment. As a major source of LAB, the study highlights the significance of fermented foods, supporting the notion that dietary decisions have a direct influence on the composition of the gut microbiota and general health (Pasolli et al. 2020). Following allo-HCT, lactic acid is essential for controlling the GVL impact. Lactic acid, which was once thought to be a metabolic waste product, has recently been demonstrated to have immunomodulatory qualities. Lactic acid specifically has the ability to regulate T cell activation and differentiation, hence enhancing anti-leukemic responses and inhibiting excessive inflammatory responses that result in GVHD. Because of its dual function, lactic acid may be a therapeutic target to improve outcomes for patients receiving allo-HCT. This means that methods to increase lactic acid production or its effects may help strike a balance between less tissue damage from GVHD and efficient leukemia control (Uhl et al. 2020).

### Lipocalin-2 (LCN2) and acute GVHD

The importance of the gut microbiota in regulating the severity of aGVHD has been brought to light by various studies (Koyama et al. 2023). Using single-cell RNA sequencing, a recent study found that mice with intestinal aGVHD had a population of neutrophils that expressed lipocalin-2 (LCN2). Recombinant LCN2 therapy or the transfer of LCN2-overexpressing neutrophils decreased the severity of aGVHD, but the absence of hematopoietic or epithelial LCN2 increased the severity of aGVHD and changed the microbiome. By means of the LCN2 receptor SLC22A17, LCN2 mechanistically triggered insulin-like growth factor 1 receptor (IGF-1R) signaling in macrophages, which raised the production of interleukin-10 (IL-10) and decreased the expression of major histocompatibility complex class II (MHCII). Transferring macrophages prepared with LCN2 decreased the severity of aGVHD, but it had no effect on graft-versus-leukemia. Moreover, LCN2 enhanced IGF-1R signaling in human macrophages and was associated with IL-10 production in intestinal biopsies in several cohorts of aGVHD patients. In summary, the study found an intestinal neutrophil population that expressed LCN2, which decreased the severity of aGVHD by improving macrophage IL-10 production and lowering MHCII expression (Czech et al. 2024).

## GM intervention with GVHD

Previous studies have indicated that gut microorganisms can potentially be a viable focus for treating GVHD in allo-HSCT patients. Therapeutic approaches focusing on gut microbes involve providing the necessary nutrients, choosing beneficial bacteria, administering antibiotics, and performing fecal microbiota transplantation (FMT).

### Nutritional support

Patients undergoing allo-HSCT may experience side effects such as nausea, loss of appetite, and inflammation of the oral mucosa following chemoradiotherapy, leading to a decrease in oral intake of nutrients. Parenteral nutrition (PN) can result in intestinal flora infection and malnutrition (Zama et al. 2020). Research has indicated that providing enteral nutrition (EN) during pulp clearing can improve the likelihood of successful transplantation by reducing mortality rates and the occurrence of acute graft-versus-host disease (aGVHD) (Iyama et al. 2021). Nevertheless, EN's primary objective should be to replenish the advantageous microorganisms in the intestines effectively. We examined 20 pediatric patients who underwent hematopoietic stem cell transplantation (HSCT) (D’Amico et al. 2019). They discovered that children who received parenteral nutrition (PN) before transplantation had lower levels of short-chain fatty acids (SCFA) than those who received PN after transplantation. In contrast, patients given enteral nutrition (EN) following transplantation experienced faster restoration of their gut microbiota and exhibited higher levels of short-chain fatty acid (SCFA) production, including Ruminococcus brucei and Clostridium praxis. Nutritional supplementation also affects bloodstream infection (BSI). Researchers have uncovered evidence suggesting that the incidence of bloodstream infections (BSI) was significantly reduced in patients who received allo-HSCT along with enteral nutrition (EN) (Ciernikova et al. 2021). Although PN is commonly used in patients undergoing alloHSCT, increasing research suggests that supplementation with EN is superior.

### Resistant starch and probiotics

Beneficial bacteria flourish when probiotics are taken, which lowers the incidence and severity of GVHD. Prebiotics enhance the growth of beneficial bacteria, resulting in improved microbial diversity and reduced infection rates (Pandey et al. 2015; Sanders et al. 2019). Restoring a healthy microbiome composition is the goal of fecal microbiota transplantation, which is linked to decreased GVHD, enhanced engraftment, and increased overall survival (Ojeda et al. 2016; Pession et al. 2021). Probiotics are either a single strain or a combination of strains that can be consumed orally to enhance the balance of microorganisms in the intestines and improve intestinal stability. Various studies have demonstrated that probiotics can enhance and control the composition of microorganisms in the intestine to exert anti-inflammatory effects. In mice models of allo-HCT, a single strain of Bacteroides fragilis has been demonstrated to preserve gut integrity and lessen the severity of GVHD. The administration of this strain by oral gavage greatly reduced the development of both acute and chronic GVHD by promoting beneficial commensal bacteria and increasing the diversity of the gut microbiota. Bacteroides fragilis may be essential for preserving intestinal health and reducing GVHD in transplant recipients, according to these data (Sofi et al. 2021). Adding Lactobacillus rhamnosus to mice undergoing allo-HSCT can decrease inflammation and the likelihood of bacterial infection. Consequently, aGVHD in mice is reduced after allo-HSCT, enhancing their survival rate. Simultaneously, probiotic Lactobacillus plantarum demonstrated favorable safety and effectiveness in children and adults who underwent allo-HSCT. A Phase III clinical trial is currently in progress (NCT03057054) to investigate the efficacy of oral Lactobacillus plantarum in preventing and treating gastrointestinal GVHD (Chang et al. 2021; Sanders et al. 2019). Treatment approaches for HSCT that address microbiota dysbiosis are shown in Table 3.

**Table 3:** Treatment approaches for HSCT that address microbiota dysbiosis

Techniques for therapy	Anticipated result	Action mechanism	References
Probiotics	Decreased occurrence and severity of GVHD	Encourage growth of beneficial microorganisms	Yoshifuji et al. (2020)
Prebiotics	Boost the development of helpful microorganisms	Enhanced microbial diversity and decreased incidence of infection	Serrano-Villar et al. (2017)
Antibiotic stewardship	Preserved diversity of microorganisms and decreased likelihood of problems	Reduce the usage of unneeded broad-spectrum antibiotics	Habibi and Rashidi (2023), Zeiser et al. (2023)
Fecal microbiota transplantation	Lower GVHD, better overall survival, and enhanced engraftment	Restore a balanced makeup of microbiomes	Ojeda et al. (2016)

### Prebiotics

A few prebiotics that have demonstrated considerable promise in preventing and reducing GVHD in patients after HCT include resistant starch (RS), fructo-oligosaccharides (FOS), galacto-oligosaccharides (GOS), and inulin. Prebiotic resistant starch ferments in the colon rather than being broken down in the small intestine, producing healthy SCFAs like butyrate (Sofi et al. 2021) In addition to being essential for gut health and epithelial integrity, butyrate promotes the growth of Tregs, which are essential for controlling GVHD because they can inhibit immunological activation. Another study shed light on the resistant potato starch (RPS) role on intestinal microbiome and metabolites, including the short-chain fatty acid butyrate. In 10 adults receiving allogeneic hematopoietic stem cell transplantation (HCT), the study evaluated the viability of using RPS as a dietary intervention. RPS was taken daily by the participants from day-7 to day 100 post-transplant. The study discovered considerably higher fecal butyrate levels with RPS ingestion (P < 0.0001) and attained over 60% adherence. The profiles of plasma metabolites on RPS were more stable than those on historical controls, particularly during engraftment (P < 0.05), even though they changed independently of RPS. Based on these findings, RPS is a viable intervention that has a major effect on intestinal and plasma metabolites in recipients of HCT (Riwes et al. 2023) Another prebiotic that boosts SCFA synthesis and encourages the development of a healthy gut bacteria is Fructo-Oligosaccharides (FOS). Studies have shown that FOS supplementation increases the diversity of gut microbes and moves microbial populations in favor of those that generate SCFAs, which may help patients undergoing HCT control their immunological responses. Through altering the gut microbiota's composition and maybe increasing Treg activity, FOS has been demonstrated to lower the incidence of GVHD (Andermann et al. 2021). The potential of Galacto-Oligosaccharides (GOS) to promote the development of gut-dwelling bifidobacteria, which have been linked to anti-inflammatory properties, has been investigated. Early results indicate that GOS helps prevent GVHD by maintaining microbial diversity and increasing gut barrier function, which lowers inflammation. Clinical trials are investigating GOS's involvement in modifying the gut microbiota and enhancing immune responses following HCT (Stein-Thoeringer 2022). Like other soluble fibers, inulin is a prebiotic that boosts the generation of SCFA and encourages the growth of beneficial bacteria. Inulin has been shown in studies to help maintain a balanced gut flora and reduce mucosal damage during HCT. Because of its ability to boost the immune system and maybe lessen the severity of GVHD, it is a good option for dietary treatments in patients undergoing HCT (Corrêa et al. 2023).

### Antibiotics

Antibiotics are routinely administered as a preventive measure against bacterial infections in the allo-HSCT regimen because transplant problems account for 20–60% of HSCT patients who advance to systemic infection (Akhmedov and Espinoza 2024). Research has demonstrated that the use of wide-ranging antibiotics, such as imipenem-cilastatin or piperacillin-sulbactam, in clinical settings can increase the occurrence of GVHD in the lower gastrointestinal tract. The allo-HSCT mice exhibited an increase in ackermania mucus and a decrease in Clostridium bacilli following the administration of these medications. Nevertheless, patients who received narrow-spectrum antibiotics, such as amtraxam or cefepime, showed no discernible impact. These findings indicate that symbiotic bacteria resistant to certain antibiotics can serve as indicators of GVHD (Josefsdottir et al. 2017). Research has demonstrated that different gut microbes are associated with the clinical outcomes of allo-HSCT. For example, a higher prevalence of cyanobacteria suggests a higher likelihood of survival, whereas a decrease in cyanobacteria is typically associated with the proliferation of potentially harmful bacteria. There is a relationship between the proliferation of Escherichia coli and the emergence of GVHD in mice. They also observed that oral administration of polymyxin B may effectively decrease Escherichia coli and ameliorate GVHD. Recent research has indicated that Enterococcus is linked to an unfavorable prognosis and can significantly decrease overall survival, as well as the likelihood and progression of acute GVHD. Furthermore, thorough intestinal purification using potent non-absorbable antibiotics can effectively prevent the movement of intestinal bacteria and their substances, decrease the signal from pattern recognition receptors, and mitigate inflammation and immune system activation. Consequently, this approach significantly decreases the likelihood of developing acute graft-versus-host disease (aGVHD) following allo-HSCT. Research has indicated that patients who use rifaximin experience a reduced occurrence of enterococcal bacteria after transplantation. Additionally, they have a higher concentration of urinary 3-indole sulfate and a lower mortality rate within one year of transplantation. Furthermore, their overall survival rate was higher. These findings suggest that enterococcal flora may have detrimental regulatory effects. Enterococcus faecalis is a constituent of the Enterococcus group, and it can impact the mucosal barrier using metalloproteinases and contribute to inflammation in the intestines. Hence, choosing suitable antibiotics for particular bacteria, such as Enterococcus, can affect the gut microbiota and overall survival following allo-HSCT[57]. Subsequent research has demonstrated that plasma protein biomarkers, namely St2, TnFR1, and REG3α, might be noninvasive diagnostic and predictive tools for aGVHD (Kakihana et al. 2016). Additionally, higher expression of these biomarkers is associated with decreased microbial diversity. Hence, the correlation between GVHD biomarkers and gut microbiota could be a foundation for developing future therapeutic approaches.

## Understanding HLA and its role in aGVHD

The human leukocyte antigen (HLA) difference between the donor and the recipient is one of the main causes of aGVHD. Here we discuss the essential relationship between HLA difference and the onset and severity of aGVH. Since HLA molecules are necessary for differentiating between self and non-self-tissues, they are vital to the immune system. The way peptide antigens are presented to T cells, which triggers an immunological response, is significantly influenced by HLA molecules (Rojas et al. 2024). When the T cells from a donor recognize the recipient's HLA as foreign, it can lead to aGVHD. The primary cause of this response is the identification of a mismatch between the donor and recipient's key HLA antigens (HLA-A, HLA-B, HLA-C, HLA-DR, HLA-DQ, and HLA-DP) (Tran et al. 2024).

### The induction mechanism of aGVHD

The process starts when recipient antigen-presenting cells (APCs) that express non-self HLA molecules activate allogeneic donor T cells that were delivered through the graft. This exchange is known as “allorecognition.” After recognizing these differences, T cells multiply and develop into effector T cells, which target the recipient's organs and tissues with precision. The skin, liver, and gastrointestinal tract are critical target organs that result in aGVHD symptoms such skin rashes, liver failure, and gastrointestinal problems (Malard et al. 2023).

### The association between HLA disparity and aGVHD phenotypes

Diverse aGVHD phenotypes may result from HLA variability. In contrast to mismatch in other HLA class II antigens, research has demonstrated that mismatch at HLA-DP causes more noticeable gastrointestinal involvement (Arrieta-Bolaños 2024).Thus, knowing the different kinds of HLA mismatches can help forecast the aGVHD phenotype more accurately, which is essential for creating mitigation plans. Since there is no doubt that HLA discrepancy and aGVHD are related, several methods have been used to reduce this risk. By eliminating the cells that cause the alloreactive reaction, for instance, T cell-depleted grafts can lower the risk of aGVHD (Xu et al. 2024) Furthermore, treatments such post-transplant cyclophosphamide have been investigated; these work to control T cell activation and proliferation, which lowers the risk of aGVHD even in cases where there is some degree of HLA mismatch (Olivieri and Mancini 2024).

## FMT

FMT involves the transfer of a fecal suspension obtained from a healthy donor into a patient's gastrointestinal tract. Its main applications in allo-HSCT patients are (1) treating recurrent Clostridium difficile infections, (2) treating cortisol-resistant or dependent acute graft-versus-host disease (aGVHD), (3) preventing microbial damage during transplantation, and (4) treating intestinal colonization by multidrug-resistant bacteria (MDRB).Research has indicated that between 15 and 30% of individuals who undergo allo-HSCTexperience an infection known as Clostridium difficile infection (CDI) during the transplantation process. Treating recurrent CDI is challenging because of the frequent ineffectiveness of antibiotic therapy. The infusion of donor stool into the duodenum not only relieves the symptoms of recurrent Clostridium difficile infection (CDI) but also enhances bacterial variety by increasing the abundance of Bacillus and Clostridium clusters IV and XIVa while decreasing the presence of Proteus species. Koneru et al. conducted fecal microbiota transplantation (FMT) in patients undergoing allo-HSCTwho had intestinal GVHD that was refractory or dependent on cortisol (Koneru et al. 2023). All patients responded positively to FMT, with no reported adverse effects directly linked to FMT. We analyzed a cohort of 15 individuals who experienced refractory intestinal GVHD following allo-HSCT therapy. The patients were treated with fecal microbiota transplantation (FMT). Gastrointestinal symptoms and diarrhea improved in 10 patients, and the balance of the gut microbiota was restored. The rise in multidrug-resistant bacteria (MDRB) poses a significant challenge to current treatment options. It significantly increases the risk of therapy in patients undergoing allo-HSCT. Research has indicated that fecal microbiota transplantation (FMT) is a safe treatment for patients with secondary multidrug-resistant bacteria (MDRB). No serious adverse effects were observed in the patients, except for constipation in one patient and mild diarrhea in two. Of the ten patients who underwent allo-HSCT, 7 successfully established gut bacteria at an average follow-up period of 13 months (ranging from 4 to 40 months). Another outcome of FMT treatment for multidrug-resistant bacteria (MDRB) in two patients who underwent alloHSCT revealed that one patient accomplished engraftment of genetically modified (GM) cells without any problems. However, the other study did not produce a therapeutic outcome (Biernat et al. 2020).

Although numerous studies have demonstrated the remarkable preventative and therapeutic effects of FMT on allo-HSCT, the precise differentiation of microorganisms in recipients following FMT remains uncertain (Nguyen et al. 2021). Second-generation sequencing technology was used to investigate the longitudinal dynamics of intestinal bacteria, fungi, and viruses in patients with GVHD receiving numerous FMT treatments. These findings indicate that the intestinal bacterial, fungal, and viral communities exhibited distinct reactions to FMT. Additionally, the intestinal bacterial populations in GVHD patients showed gradual restoration following numerous FMT interventions. The level of variety exhibited an initial increase followed by a subsequent drop in the fungal colonies. Viral makeup undergoes alterations following multiple fecal microbiota transplants (FMTs), but diversity levels consistently increase. These results are essential for future studies on the mechanism of FMT treatment after allo-HSCT for GVHD and can enhance the safety and effectiveness of FMT.

These investigations indicate that reversing intestinal dysbiosis using FMT can prevent and enhance intestinal GVHD. Nevertheless, there is a need to strengthen various elements of FMT therapy, including the method of administration (enema, capsules, and nasal duodenal infusion), timing, frequency, and selection of donor source (autologous, related, or healthy donor). Furthermore, conducting screenings of FMT donors to identify risks, such as drug-resistant bacteria, which can lead to bloodstream infections originating from FMT, can significantly enhance the prevention of potential dangers.

### FMT trials in the post-HCT setting in GVHD prevention and treatment

With a focus on preventing and treating GVHD, a number of clinical trials have investigated the application of FMT in the HCT context. The noteworthy studies listed below demonstrate how FMT's function in treating GVHD is changing.

#### FMT phase II trial for acute GVHD prevention

In a randomized phase II trial, the effectiveness of oral, encapsulated, third-party FMT in avoiding acute GVHD in patients receiving allogeneic HCT was examined in comparison to a placebo. The study concentrated on when to administer FMT (i.e., after neutrophil recovery). A successful donor microbiota engraftment was linked to a lower incidence of aGVHD, according to preliminary findings, and individuals who showed greater microbial variety benefited from the treatment (Rashidi et al. 2023).

#### Autologous FMT for the prevention of GVHD

The effectiveness of autologous FMT (using a patient's own stool) and a control group in the early post-transplant phase were examined in this prospective randomized study. According to the results, the autologous FMT group saw a considerably decreased incidence of acute GVHD (22.2% versus 42.5% in the control group) Furthermore, overall survival rates increased, indicating that auto-FMT may be an effective way to reduce the risk of GVHD (Dougé et al. 2023).

#### FMT for steroid-refractory acute GVHD

FMT's effectiveness in treating individuals with steroid-refractory acute GVHD was evaluated in this pilot research. The experiment found that while some patients suffered problems like bacteremia, a large percentage of treated individuals achieved clinical improvement after receiving FMT from unrelated donors. This study shed light on FMT's potential for refractory cases—those in which conventional treatments are unsuccessful (Liu et al. 2022).

#### Third-Party FMT with corticosteroids

Promising response rates were seen in a trial that treated individuals with acute GVHD of the lower gastrointestinal tract utilizing third-party FMT in conjunction with corticosteroid therapy. The initial findings indicated that combining these therapies improved outcomes, thus emphasizing the need for more comprehensive approaches in high-risk patient (DeFilipp et al. 2024).

#### Multi-center FMT study

FMT's effectiveness in treating patients with gastrointestinal GVHD was assessed in a multi-center trial. Patients from multiple treatment facilities participated in this study to determine its wider applicability and efficacy, and in certain instances, the results were better than those of traditional therapy (Kujawska et al. 2024).

#### FMT for GVHD and infections after transplantation

A study aimed to assess FMT's capacity to prevent infections in conjunction with the treatment of aGVHD. Together with the usual GVHD prophylaxis, some subjects received FMT to restore gut microbiota. The results highlighted the advantages of controlling microbial diversity and lowering the risk of infection (Kakihana et al. 2016).

#### FMT’s effectiveness and safety in allogeneic HCT

Researchers investigated the safety and effectiveness of standardized third-party FMT following allogeneic HCT in a large-scale experiment. In a patient population more susceptible to infections and problems following HCT, this study gained notoriety by establishing the baseline safety profile for FMT. The study demonstrated how FMT may improve gut microbiota diversity and have a beneficial impact on GVHD outcomes (Habibi and Rashidi 2023).

### Impact of antibiotics on FMT

Restoring a healthy gut microbiota through FMT is very useful in treating recurrent Clostridium difficile infections and may also help with other intestinal and systemic illnesses. However, the efficacy of FMT might be greatly impacted by the use of antibiotics in individuals undergoing the procedure.

#### Disruption of microbial engraftment

The engraftment of donor microbiota, which is essential for the transplant's effectiveness, can be disrupted by antibiotic use soon after FMT (Allegretti et al. 2018). Antibiotics can alter the intestinal microbiota both quantitatively and qualitatively, which alters the variety and makeup of the gut flora and causes this disturbance (Dethlefsen and Relman 2011; Ianiro et al. 2016).

#### Pre-treatment with antibiotics

Pretreatment with antibiotics prior to FMT has a complicated and situation-specific role. Although the overall effect on donor microbiota engraftment is minimal, pre-treatment with antibiotics has occasionally been demonstrated to deplete the current microbiota, which may be advantageous for the engraftment of specific donor-derived taxa, such as Bifidobacterium (Freitag et al. 2019) Patients with ulcerative colitis who get antibiotics prior to FMT have better bacterial dysbiosis and greater clinical remission rates, which may increase the effectiveness of FMT in some circumstances (Ishikawa et al. 2016).

#### Microbiome recuperation following antibiotic use

FMT has been shown to restore the makeup and diversity of the microbiome after antibiotic therapy. The richness and diversity of bacterial species can be significantly reduced by antibiotics, which FMT can assist to reverse (Le Bastard et al. 2018). FMT has been demonstrated to restore the pre-treatment microbiome profile and recover particular taxa that have been depleted in animal models. This is important for halting the growth of harmful bacteria and preserving a healthy gut environment (Le Bastard et al. 2018).

#### Mechanisms of interaction with FMT

Prior to FMT, antibiotic pretreatment may alter the recipient's gut microbiota makeup and intestinal environment. This alteration may lessen the transplanted fecal microbiota's engraftment, which is essential to the treatment's efficacy. In contrast to those who underwent FMT without prior antibiotic treatment, studies have demonstrated that patients who received antibiotics prior to FMT had noticeably reduced rates of donor microbiota engraftment (Singh et al. 2022).

#### Antibiotic types and their impacts

The outcome of FMT may also be significantly influenced by the particular kind of antibiotic employed. In contrast to narrow-spectrum antibiotics, the use of broad-spectrum antibiotics may have a more significant effect on gut flora, increasing the likelihood of dysbiosis and decreasing the efficacy of FMT Antibiotics like metronidazole and ciprofloxacin have been demonstrated to adversely affect the microbiota, which may jeopardize the donor's microbial variety and capacity to establish a healthy gut environment after transplantation.

In summary, whereas antibiotics can hinder FMT by interfering with microbial engraftment, their judicious pre-treatment use under specific circumstances may improve FMT effectiveness. Additionally, FMT is an essential tool for reestablishing the variety of the gut microbiota after antibiotic treatment, underscoring its promise in clinical contexts where antibiotics are required.

## Conclusion and future prospective

GVHD is the primary cause of mortality in patients undergoing hematopoietic stem cell transplantation (HSCT). The microbial flora and its metabolites regulate the immune system and maintain balance in the gut environment. Consequently, they may influence host vulnerability to GVHD. Several studies have demonstrated that allo-HSCT can harm the intestinal mucosa, reducing the diversity of symbiotic microbes and causing an imbalance in intestinal flora. Research has indicated that the intestinal flora's nature significantly impacts bloodstream infection, graft-versus-host disease, organ toxicity, and overall survival following allo-HSCT. With the ongoing advancement of technology, researchers have developed a more comprehensive understanding of the precise function of the gut microbiota in the pathogenesis of GVHD. The specific elements of the microbiota that worsen or improve risk factors for acute graft-versus-host disease (aGVHD) are unknown. However, patients with a higher presence of Lactobacillus, Clostridium, probiotics, Brucella, Bacteroides, and Clostridium groups IV and XIVa showed a reduced incidence of GVHD. Administering these strains to patients with GVHD can help prevent the occurrence of GVHD.However, the increased presence of Enterococcus, Proteus, and Bifidobacterium can worsen GVHD.Research has demonstrated that antibiotics during allo-HSCT lead to notable alterations and reduce the variety of microorganisms in the gut microbiota.When the variety and stability of the intestinal microbiota are disrupted, disease-associated organisms are taken over, ultimately leading to GVHD. Nevertheless, additional research is required to validate and enhance these findings.

Research has demonstrated a correlation between microbiota alterations and worsening GVHD in patients with GVHD. This correlation may be attributed to the harm caused to the intestinal mucosa, particularly the destruction and depletion of Paneth and goblet cells. Thus, enhancing the viability or restoration of epithelial cells may impede the excessive growth of pathogens and the dissemination of MAMPs, PAMPs, and danger-associated molecular patterns (DAMP). Microbial metabolites, including butyrate, supercritical fatty acids, and indole, are markers of the impact of microbial flora on graft-versus-host disease. These metabolites can control the function of the mucosal barrier and the expression of genes associated with inflammation, both pro-inflammatory and anti-inflammatory. Nevertheless, exploring these microbial metabolites as therapeutic approaches has yet to be well-researched. Fecal microbiota transplantation (FMT) has emerged as a potent strategy for effectively treating severe clostridioides difficile infection (CDI). Advancements in understanding the function of gut microbiota in graft-versus-host disease will facilitate progress in the field and establish a scientific foundation for developing novel strategies to prevent and cure post-transplant GVHD. Examining the alterations in the bacterial microbiota during GVHD is crucial, and it is essential to study the composition of the microbiota and virions specifically. Studying colony interactions is critical because the host microbiome consists of diverse colonies. Research on the functional genomics of host microorganisms can provide a more detailed understanding of the complicated interactions between different colonies, which can then be used to develop targeted preventative and treatment strategies.

## Data Availability

No datasets were generated or analysed during the current study.
